# Structural Basis for Mitotic Centrosome Assembly in Flies

**DOI:** 10.1016/j.cell.2017.05.030

**Published:** 2017-06-01

**Authors:** Zhe Feng, Anna Caballe, Alan Wainman, Steven Johnson, Andreas F.M. Haensele, Matthew A. Cottee, Paul T. Conduit, Susan M. Lea, Jordan W. Raff

**Affiliations:** 1 The Sir William Dunn School of Pathology, University of Oxford, South Parks Road, Oxford OX1 3RE, UK

**Keywords:** centrosome, centriole, PCM, Centrosomin, Cnn, Plk1, mitosis

## Abstract

In flies, Centrosomin (Cnn) forms a phosphorylation-dependent scaffold that recruits proteins to the mitotic centrosome, but how Cnn assembles into a scaffold is unclear. We show that scaffold assembly requires conserved leucine zipper (LZ) and Cnn-motif 2 (CM2) domains that co-assemble into a 2:2 complex in vitro. We solve the crystal structure of the LZ:CM2 complex, revealing that both proteins form helical dimers that assemble into an unusual tetramer. A slightly longer version of the LZ can form micron-scale structures with CM2, whose assembly is stimulated by Plk1 phosphorylation in vitro. Mutating individual residues that perturb LZ:CM2 tetramer assembly perturbs the formation of these micron-scale assemblies in vitro and Cnn-scaffold assembly in vivo. Thus, Cnn molecules have an intrinsic ability to form large, LZ:CM2-interaction-dependent assemblies that are critical for mitotic centrosome assembly. These studies provide the first atomic insight into a molecular interaction required for mitotic centrosome assembly.

## Highlights

• The conserved PReM and CM2 domains of Cnn co-assemble into micron-scale structures
• The crystal structure of the PReM-LZ:CM2 complex is solved to 1.82 Å
• Mutations that block PReM-LZ:CM2 assembly in vitro block centrosome assembly in vivo
• Phosphorylation of PReM by Polo/Plk1 promotes scaffold assembly in vitro and in vivo

## Graphical Abstract

**Figure:**
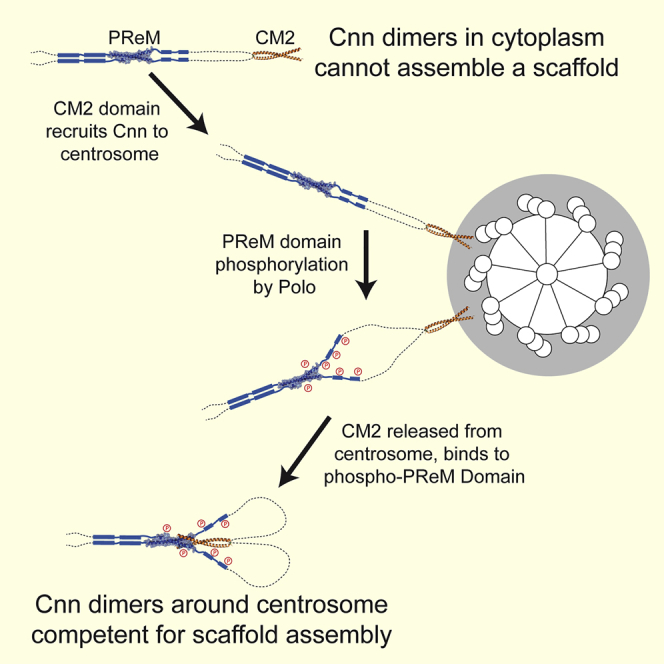

## Teaser

Structural and biochemical analyses paint a new picture of how the pericentriolar material forms micron-scale structures that recruit PCM and nucleate microtubules.

## Introduction

Centrosomes play an important part in many cell processes, and they are formed when pericentriolar material (PCM) is recruited around the mother centriole (Conduit et al., 2014b, Wang et al., 2011). Several hundred proteins are thought to be concentrated in the PCM, and these include proteins involved in nucleating and organizing microtubules (MTs) as well as many important cell-cycle regulators and signaling molecules (Conduit et al., 2015, Vertii et al., 2016). It is unclear, however, how the hundreds of proteins localized within the PCM assemble into a functional organelle.

During interphase, the centrioles in many cell types organize relatively small amounts of PCM, and recent studies have revealed that the interphase PCM is spatially organized (Fu and Glover, 2012, Lawo et al., 2012, Mennella et al., 2012, Sonnen et al., 2012). In particular, Pericentrin—the Pericentrin-like-protein (PLP) in flies—is asymmetrically distributed within the interphase PCM, with its C terminus close to the mother centriole and its N terminus stretched outward away from the centriole. The interphase PCM is largely assembled within the boundary defined by Pericentrin, which is required to recruit the interphase PCM (Lawo et al., 2012, Mennella et al., 2012).

As cells prepare to enter mitosis, there is a dramatic increase in the amount of PCM recruited around the centrioles (Conduit et al., 2015, Palazzo et al., 2000). The mitotic PCM appears less well organized than the interphase PCM, and studies in flies suggest that Centrosomin (Cnn) plays a crucial part in assembling the expanded mitotic PCM (Conduit et al., 2010, Lucas and Raff, 2007, Megraw et al., 2001, Megraw et al., 1999, Vaizel-Ohayon and Schejter, 1999). In fly embryos, Cnn is recruited around mother centrioles in an Spd-2-dependent manner, where it gets phosphorylated by Polo, allowing it to assemble into a scaffold structure that then fluxes outward away from the mother centriole along the centrosomal MTs (Conduit et al., 2014b, Conduit et al., 2014a). Cnn and Spd-2 cooperate to recruit other proteins to mitotic centrosomes, and, in the absence of both proteins, mitotic centrosome assembly is abolished (Conduit et al., 2014b).

In worms, SPD-5 is the likely functional ortholog of Cnn, although the two proteins are not obviously related by sequence. SPD-5 is essential for mitotic PCM assembly, and, like Cnn, it is also recruited to centrioles by SPD-2, where it becomes phosphorylated by PLK-1, allowing SPD-5 to assemble into a scaffold structure that recruits proteins to the mitotic centrosome (Hamill et al., 2002, Kemp et al., 2004, Pelletier et al., 2004, Woodruff et al., 2015). Recombinantly expressed SPD-5 can assemble into micron-scale assemblies in vitro, and this process is enhanced by the presence of SPD-2 and PLK-1 (Woodruff et al., 2015, Wueseke et al., 2016). Plk1, Cep215/Cdk5Rap2, and Cep192—the vertebrate homologs of Polo/PLK-1, Cnn, and SPD-2 (there are no obvious vertebrate homologs of SPD-5), respectively—have all also been implicated in mitotic PCM recruitment (Barr et al., 2010, Choi et al., 2010, Gomez-Ferreria et al., 2007, Haren et al., 2009, Joukov et al., 2014, Lizarraga et al., 2010, Zhu et al., 2008).

Although there is increasing evidence that proteins like Cnn and SPD-5 can form scaffold-like structures that help recruit the mitotic PCM, the nature of these scaffolds remains mysterious. In particular, it is unclear whether these proteins form highly organized scaffolds with a well-defined structure based on precise molecular interactions or whether they form more amorphous interactions and structures that allow the PCM to phase-separate from the cytoplasm as a liquid- or gel-like droplet (Conduit et al., 2015, Woodruff et al., 2014, Zwicker et al., 2014). Intriguingly, no well-ordered structural interaction has yet been identified that is required for mitotic PCM assembly, lending credence to the idea that the mitotic PCM may be intrinsically unstructured.

*Drosophila* Cnn contains a phospho-regulated multimerization (PReM) domain (Conduit et al., 2014a) that consists of a previously identified leucine zipper motif (LZ) (Heuer et al., 1995) followed by a series of highly conserved Ser/Thr residues that can be phosphorylated by recombinant Plk1 in vitro. Alanine substitutions of either the conserved Leu residues of the LZ or of the ten conserved Ser/Thr residues strongly block Cnn-scaffold assembly in vivo, while substituting phospho-mimicking Glu/Asp mutations for the ten Ser/Thr residues allows Cnn to spontaneously form scaffold-like structures in the cytoplasm. The purified PReM domain forms an LZ-dependent dimer in vitro, but mutant forms with the phospho-mimicking 10D/E mutations can assemble into higher-order oligomers, suggesting that multimerization of the phosphorylated PReM domain is crucial for Cnn-scaffold assembly. Unfortunately, the nature of the interactions that drive PReM domain multimerization remain to be elucidated.

The conserved C-terminal Cnn-motif 2 (CM2) domain has been implicated in targeting Cnn-family proteins to centrosomes (Barr et al., 2010, Wang et al., 2010). Here, we show that CM2 not only helps recruit Cnn to centrosomes but also helps Cnn assemble into a scaffold. Purified CM2 forms a stable 2:2 tetramer with the purified LZ of the PReM domain, and we solve the crystal structure of the LZ:CM2 complex, revealing that helical dimers of LZ and CM2 interact in an anti-parallel fashion. This structure has a striking similarity to the tetrameric complex formed between two dimers of Homer1, a protein that forms a mesh-like matrix that is required for the assembly of the postsynaptic density (PSD) in neurons (Hayashi et al., 2009). In the full-length Cnn molecule, LZ is flanked by additional predicted helical sequences in the PReM domain, and, when mixed with CM2, the PReM domain does not form a tetramer but instead forms large micron-scale structures whose assembly is enhanced by Plk1-dependent phosphorylation. Point mutations that perturb the LZ:CM2 interaction perturb the assembly of these complexes in vitro and Cnn-scaffold assembly in vivo. Thus, Cnn molecules have an intrinsic ability to self-assemble into micron-scale structures, and this requires the well-ordered LZ:CM2 interaction interface.

## Results

### The CM2 Domain Targets Cnn to Centrosomes but Is Also Required for Efficient Cnn-Scaffold Assembly

To test the function of the Cnn-CM2 domain, we generated lines expressing WT GFP-Cnn or a form of the protein lacking the CM2 domain (GFP-Cnn-ΔCM2) in a *cnn* mutant background. In most fly somatic cells, the centrioles organize very little PCM or MTs during interphase (Jankovics and Brunner, 2006, Martinez-Campos et al., 2004, Rogers et al., 2008), but in the rapidly dividing early syncytial embryo, the centrosomes are essentially always in a mitotic-like state, maintaining an enlarged, Spd-2- and Cnn-dependent, PCM throughout these early nuclear divisions (Conduit et al., 2010, Megraw et al., 1999). In these embryos, Cnn is constantly recruited around mother centrioles and assembles into a large scaffold that fluxes outward along the centrosomal MTs, forming “flares” that break away from the periphery of the PCM (Conduit et al., 2014b, Megraw et al., 2002).

**Figure 1:**
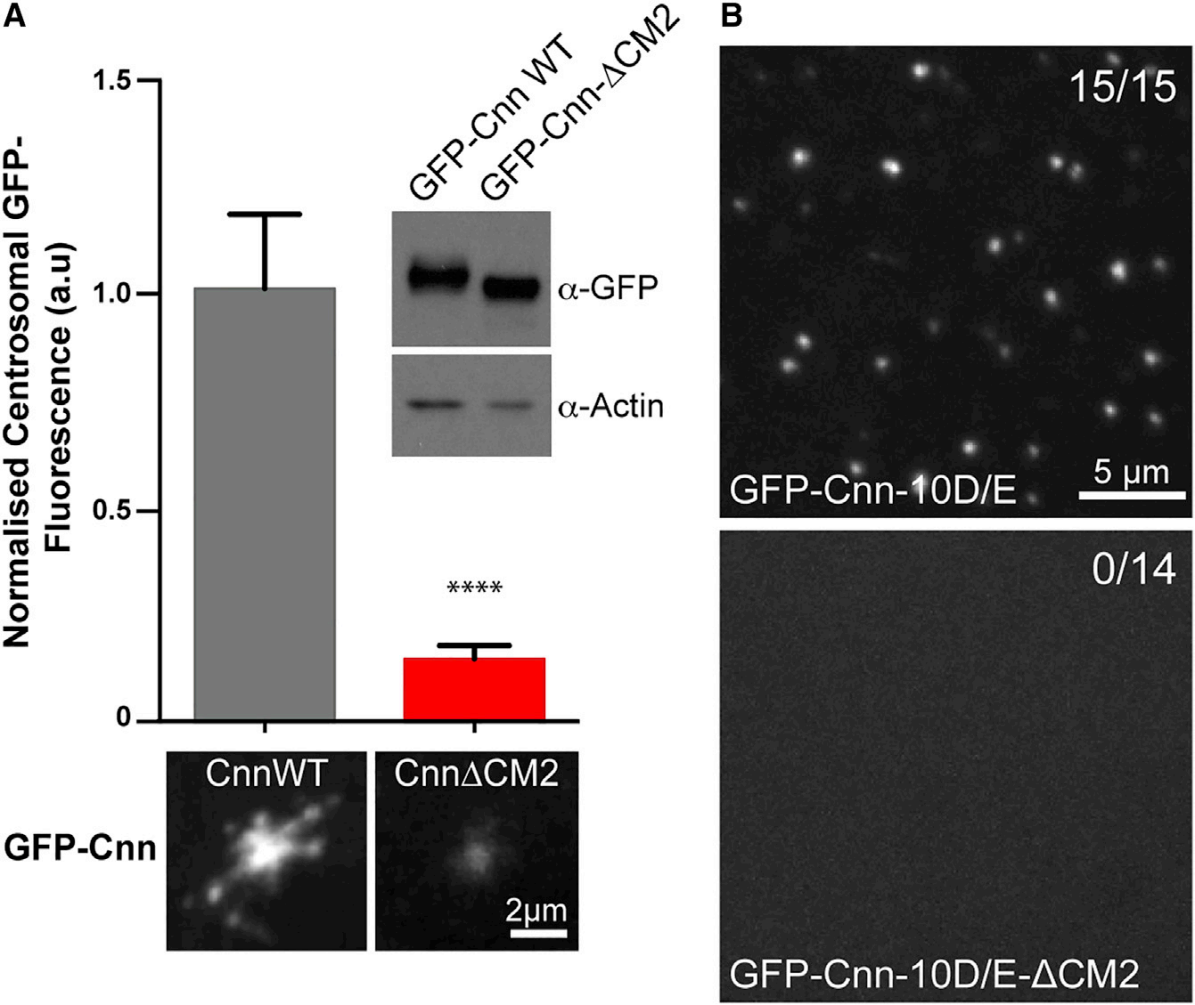
The Cnn-CM2 Domain Is Required for Centrosomal Targeting and for Scaffold Assembly (A) Micrographs illustrate and graphs quantify the mean centrosomal GFP-fluorescence levels in embryos of *cnn* mutant flies expressing GFP-Cnn or GFP-Cnn-ΔCM2. Inset shows a western blot probing the relative levels of GFP-Cnn or GFP-Cnn-ΔCM2 expressed in these embryos; actin is shown as a loading control. (B) Micrographs illustrate the spontaneous assembly of cytoplasmic Cnn scaffolds in unfertilized eggs expressing GFP-Cnn-10D/E (15/15 injected eggs); no scaffolds were detectable in eggs expressing GFP-Cnn-10D/E-ΔCM2 (0/14 injected eggs). Error bars (A) represent the SD. Statistical significance was assessed using an unpaired t test in GraphPad Prism (^∗∗∗∗^p < 0.0001). Scale bars = 2 μm (A) and 5 μm (B).

This behavior of Cnn was recapitulated by WT GFP-Cnn (Figure 1A), but GFP-Cnn-ΔCM2 was only very weakly localized at centrosomes (Figure 1A). This difference was unlikely to be due to differences in protein stability as GFP-Cnn and GFP-Cnn-ΔCM2 were expressed at similar levels in transgenic embryos (Figure 1A, inset). We conclude that CM2 is required to efficiently recruit Cnn to centrosomes—as shown previously for other Cnn-family members (Barr et al., 2010, Wang et al., 2010).

The low level of GFP-Cnn-ΔCM2 at centrosomes made it difficult to ascertain whether CM2 might also be required for Cnn-scaffold assembly. We showed previously, however, that a form of Cnn in which the ten putative phosphorylated Ser/Thr residues in the PReM domain had been mutated to phospho-mimicking Glu/Asp residues (GFP-Cnn-10D/E) could spontaneously assemble ectopic “scaffolds” in the cytoplasm independently of centrosomes and require a functional LZ for their assembly (Conduit et al., 2014a). Injecting mRNA encoding the phospho-mimicking GFP-Cnn-10D/E into WT unfertilized eggs (that lack endogenous centrosomes) led to the formation of large cytoplasmic GFP-Cnn-10D/E scaffolds, while injecting mRNA encoding a form of the protein lacking the CM2 domain (GFP-Cnn-10D/E-ΔCM2) did not (Figure 1B). These data suggest that the CM2 domain is likely required for Cnn-scaffold assembly.

### The CM2 and LZ Domains Assemble into a 2:2 Tetrameric Complex

**Figure 2:**
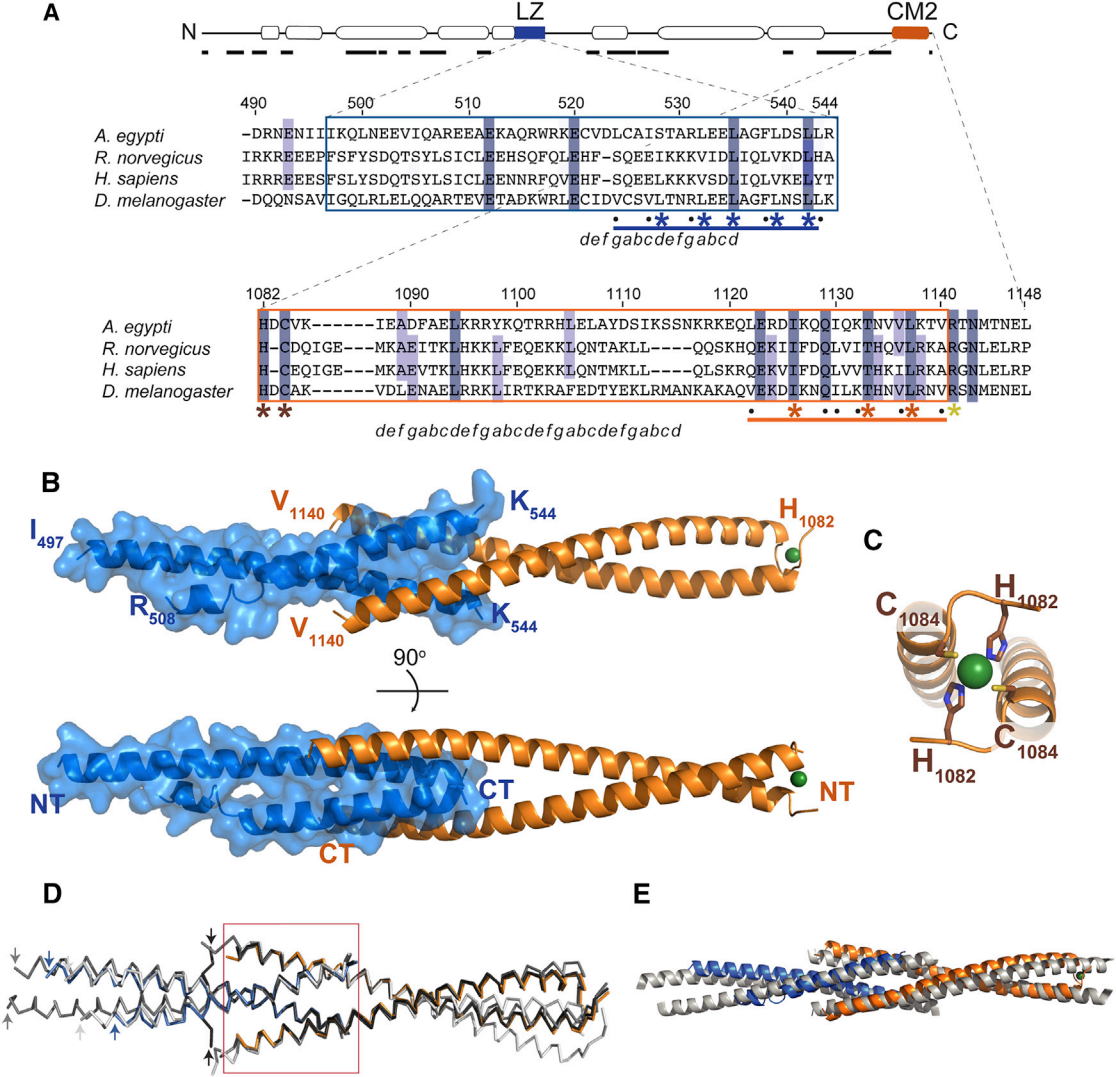
Crystal Structure of the LZ:CM2 Complex (A) Schematic illustration of *Drosophila* Cnn highlighting predicted coiled-coil regions (bubbles; predicted by COILS [Lupas et al., 1991]), predicted disordered regions (black lines; predicted by XtalPred-RF [Slabinski et al., 2007]), and the LZ (blue) and CM2 (orange) domains. Expanded regions show multiple sequence alignments (MSAs) of the regions used for crystallization (see Figure S1A for a more comprehensive MSA of the CM2 domain); boxed regions indicate residues visible in the crystal structures. Bars indicate the interaction interface with dots or asterisks over the bars highlighting residues buried in the interface. Asterisks highlight residues subjected to mutational analysis. Residues identified by SOCKET (Walshaw and Woolfson, 2001) as belonging to a canonical coiled coil in the structure are annotated beneath the sequence with *a–g* lettering. (B) Side and top views of the LZ (blue):CM2 (orange) complex, shown in cartoon representation; a space-filling model of LZ is overlaid with a reduced opacity. The coordinating Zn^*2+*^ ion is shown as a green sphere. The N terminus (NT) and C terminus (CT) of each protein are indicated. (C) Close up view of the N-terminal region of CM2 highlighting the coordination of the Zn^*2+*^ ion. (D) Ribbon diagram of three different LZ:CM2 domain crystal structures (shades of gray) overlaid on the original LZ:CM2 structure (blue and orange) shown in (B); the N termini of the different LZ constructs are indicated by arrows. The core of the LZ:CM2 interaction interface is similar in all of the structures (red box), but the surrounding helical regions exhibit considerable variation. (E) An overlay of the LZ:CM2 structure (blue:orange) and the Homer1:Homer1 tetramer (gray) (PDB: 3CVE). See also Figures S1 and S2.

**Figure 3:**
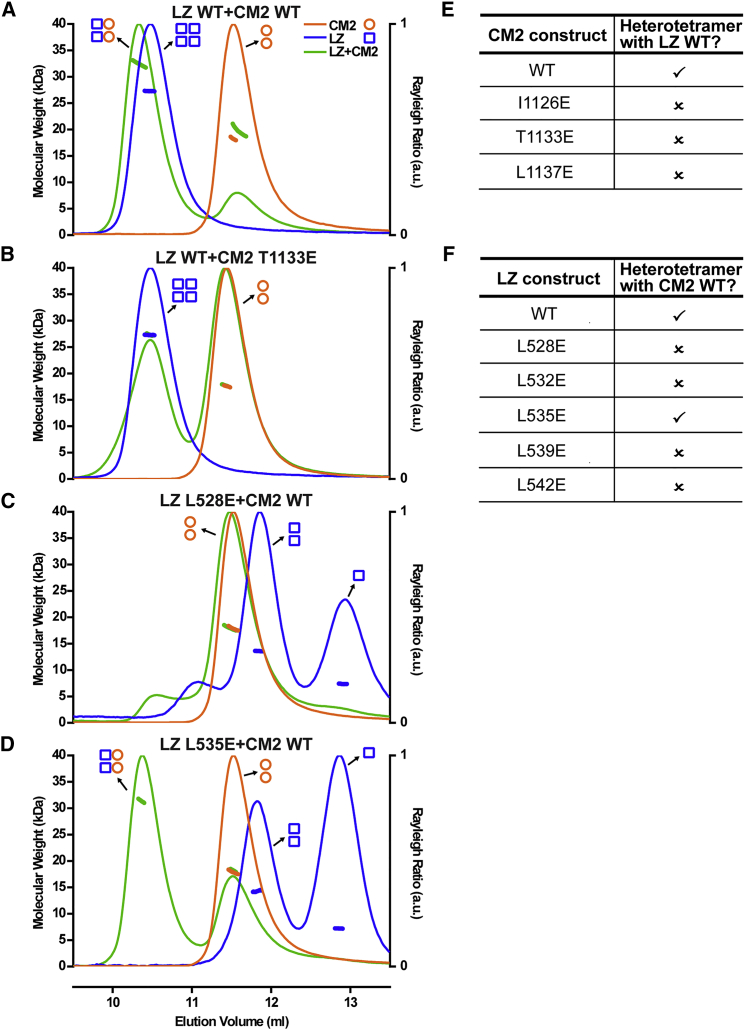
SEC-MALS Analysis of WT and Mutant LZ, CM2, and LZ:CM2 Complexes (A–D) SEC-MALS analyses of either WT LZ (blue), WT CM2 (orange), or the WT LZ:CM2 complex (green) (A), a representative example of a CM2 mutant (T1133E) that does not form a complex with LZ (B), a representative example of an LZ mutant (L528E) that does not form a complex with CM2 (C), or an analysis of the LZ mutant (L535E) that can still form a complex with CM2, even though it can no longer form a homo-tetramer on its own (D). (E and F) Tables summarizing the ability of the various CM2 mutants (E) or LZ mutants (F) to form the LZ:CM2 hetero-tetramer. See also Figures S4 and S5.

**Figure S1:**
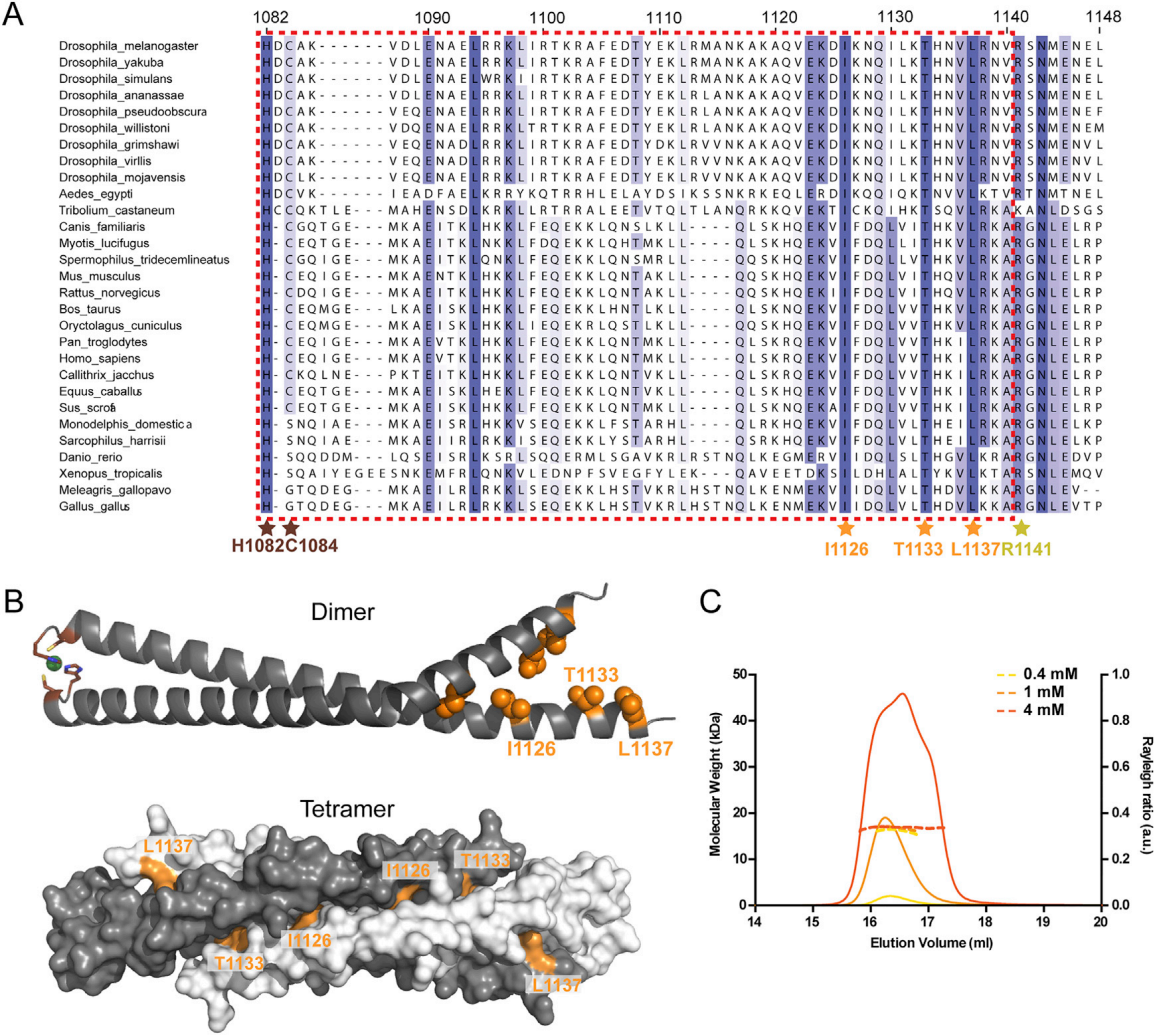
CM2 Assembles into a Helical Dimer in Solution that further Assembles into an Antiparallel Tetramer In Crystallo, Related to Figures 2 and 4 (A) An extended Multiple Sequence Alignment (MSA) of the CM2 domain: boxed residues are visible in the crystal structures. Residues that have been subjected to mutational analyses are highlighted with asterisks: color corresponds to the color illustrated in the crystal structures. (B) Cartoon representation of the CM2 dimer, and space-filling diagram of the CM2 tetramer formed *in crystallo*. Several conserved residues are highlighted in color. (C) SEC-MALS analysis of the purified CM2 domain at different concentrations, illustrating that the protein forms a stable dimer with little tendency to form higher-order oligomers even at high protein concentrations.

As our data implicated both the conserved LZ and CM2 domains in Cnn-scaffold assembly, we wondered whether they might interact. Domain boundaries were designed using PSIPRED (Jones, 1999), and we recombinantly expressed and purified a 55aa “LZ” fragment (aa490–544) and a 67aa “CM2” fragment (aa1082–1148) from bacteria (Figures 2A and S1A). SEC-MALS analysis revealed that LZ formed a stable homo-tetramer in solution, while CM2 behaved as a stable homo-dimer (Figure 3A). When the two domains were mixed in vitro, however, they reassembled to form a stable 2:2 hetero-tetrameric complex (Figure 3A).

**Figure 4:**
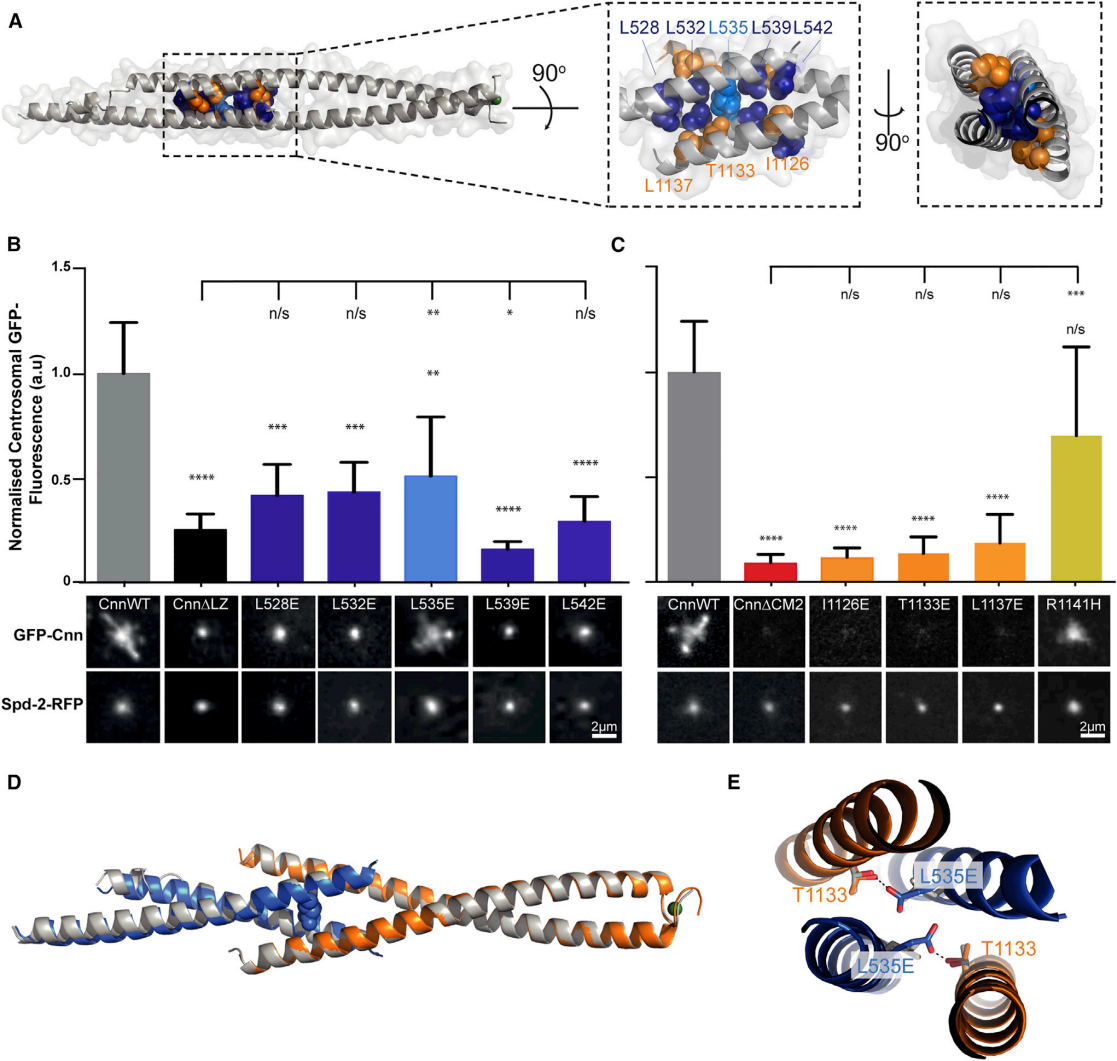
An Analysis of the Ability of Various LZ and CM2 Mutants to Support Cnn-Scaffold Assembly In Vivo (A) Views of the LZ:CM2 complex (shown in cartoon representation; space-filling model overlaid with reduced opacity) highlighting the LZ (blue) and CM2 (orange) residues in the interaction interface subjected to mutational analysis. (B and C) Micrographs illustrate and graphs quantify the centrosomal GFP-fluorescence levels of WT-GFP-Cnn or the various LZ (B) or CM2 (C) mutants; Spd-2-RFP is shown as a centrosomal marker. Note how the LZ mutants are still recruited to the centrosome, but, with the exception of the L535E, they cannot assemble a scaffold, while the CM2 mutants, with the exception of the control R1141H mutation, are not efficiently recruited to the centrosome and cannot assemble a scaffold. (D and E) Overlays of the WT LZ:CM2 complex (gray) and the LZ-L535E:CM2 complex (blue and orange). E535—shown as a space-filled residue (D)—is accommodated within the interaction interface, and the enlarged image (E) shows how E535 (blue) hydrogen bonds with the conserved T1133 residue. Error bars (B and C) represent the SD of the mean. Statistical significance (compared to WT [above each bar] and either Cnn-ΔLZ or Cnn-ΔCM2 [line at the top of the graph]) was assessed using an unpaired t test in GraphPad Prism (^∗^p < 0.05; ^∗∗^p < 0.01; ^∗∗∗^p < 0.001; ^∗∗∗∗^p < 0.0001). See also Figures S1, S2, and S3. Scale bars = 2 μm.

Crystallization of the LZ:CM2 hetero-tetramer revealed that the LZ and CM2 were entirely α helical, with each domain forming a parallel homo-dimer that interacted in an anti-parallel fashion to form the 2:2 hetero-tetrameric complex (Figure 2B; Table S1). Although the N-terminal half of CM2 formed a canonical coiled coil (Figure 2A), the C-terminal half adopted an unusual “splayed” conformation that wrapped around and clasped the coiled coil of the LZ (Figure 2B). A splayed C terminus was also observed in crystals of CM2 alone (Figure S1B; Table S1), indicating it is a feature of the CM2 sequence rather than being driven by the interaction with the LZ. Assembly of the LZ:CM2 tetramer buried the canonical Leu residues that define the LZ (L528, L532, L535, L539, L542) (blue asterisks, Figure 2A; blue residues, Figure 4A), and also three residues that appear to be invariant within CM2 domains across species (Ile1126, Thr1133, and Leu1137) (Figures 2A and S1A; orange asterisks; Figure 4A, orange residues,). We also solved the structure of several LZ:CM2 complexes that contained slightly shorter or longer versions of the LZ, again based on PSIPRED predictions (Figure 2D; Table S1). These revealed structural consistency in the four-helix bundle at the core of the interaction interface, while the coiled coils of both components that extended away from the core bundle demonstrated variability, both in terms of flexibility (angle relative to the core bundle) and the degree of order within the crystal. Thus, the LZ:CM2 interface forms a stable tetrameric core that is surrounded by more flexible dimeric helical elements.

Performing a structure-based search of the Protein Data Bank found only two similarly assembled structures (PDB: 3CVE and 4MT8), one of which is a homo-tetrameric assembly formed by the C-terminal regions of two Homer1 dimers (Figure 2E, in gray). This protein is implicated in protein-scaffold assembly within the postsynaptic density (PSD) in neurons (Hayashi et al., 2009). Intriguingly, although CM2 showed no tendency to tetramerise in solution (Figure S1C), apo-CM2 crystals contained two copies of the CM2 homo-dimer that interacted in an anti-parallel fashion to form a homo-tetramer (Figure S1B).

**Figure S2:**
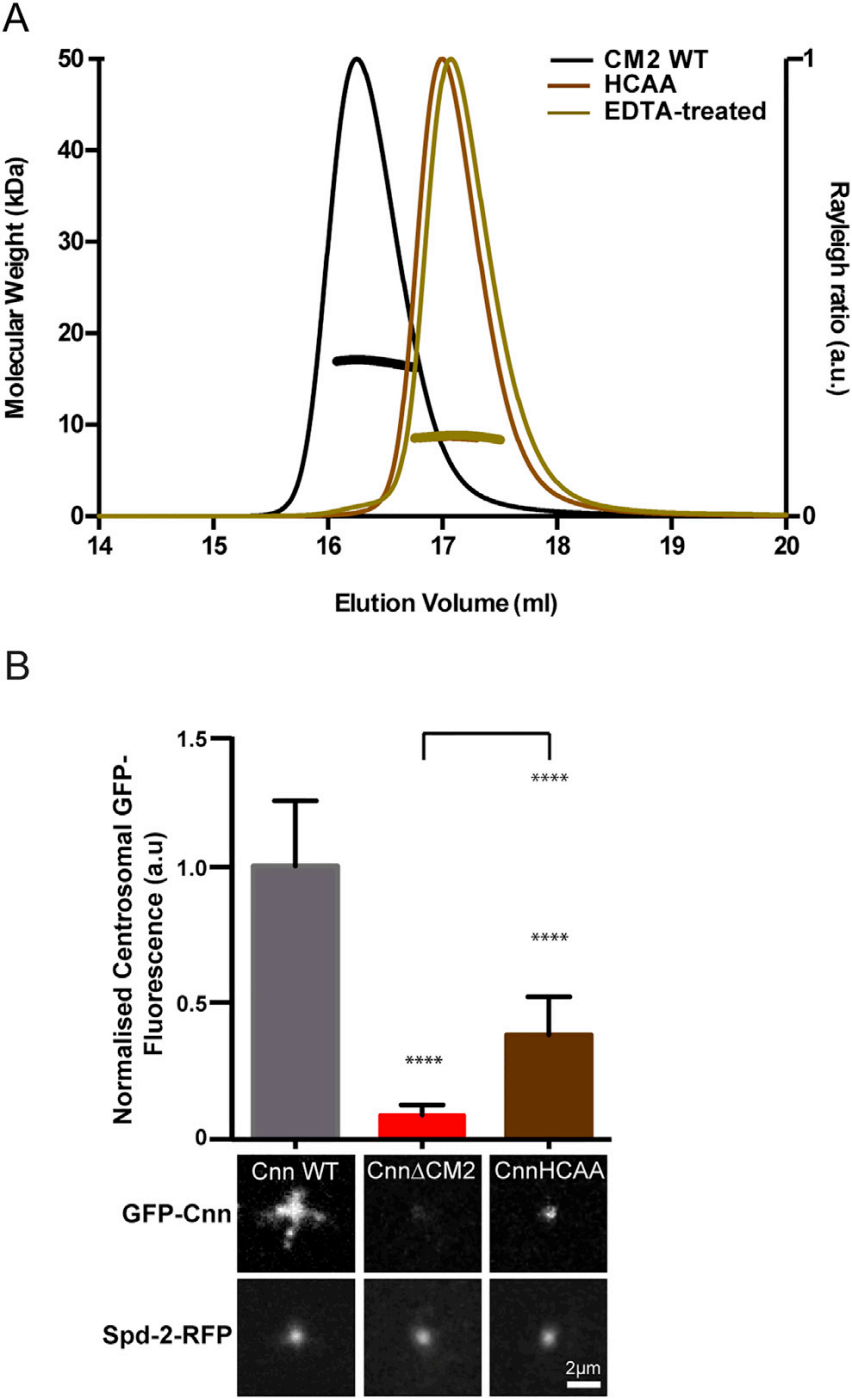
The Zn^2+^ Ion in the CM2 Dimer Is Required for CM2 Dimerization In Vitro and Efficient Cnn-Scaffold Assembly In Vivo, Related to Figures 2 and 4 (A) SEC-MALS analysis of WT-CM2 in buffer without (*black*) or with (*brown*) EDTA, or a mutant form of CM2 in which the Zn^2+^ coordinating His and Cys residues have been mutated to Ala (CM2-HCAA). Removing the Zn^2+^ or mutating the Zn^2+^ binding residues causes the purified CM2 to behave as a monomer. (B) Micrographs illustrate and graphs quantify the centrosomal localization of WT-GFP-Cnn, GFP-Cnn-ΔCM2 and GFP-Cnn-HCAA; Spd-2-RFP is shown as a centrosomal marker. Error bars represent the SD of the mean from at least 5 embryos. Statistical significance (compared to WT [above each bar] or Cnn-ΔCM2 [line at the top of the graph]) was assessed using an unpaired t test in GraphPad Prism (^∗∗∗∗^p < 0.0001). Scale bar = 2 μm.

Unexpectedly, the CM2 dimer binds a Zn^2+^ ion at its N terminus, coordinated between a His residue (H1082) that appears to be invariant in CM2 domains and a Cys residue (C1084) that is well conserved in *Drosophila* species but less so in other species (Figures 2A and S1A, brown asterisks; Figure 2C, brown residues). Zn^*2+*^ was not included in our protein preparations, so it must have been incorporated into the dimer in the bacteria and then retained throughout the purification process, indicating tight binding. Addition of EDTA to WT CM2, or mutation of the Zn^2+^-coordinating His and Cys residues to Ala (CM2-HCAA), prevented dimerization in vitro (Figure S2A), confirming that Zn^2+^ plays a structural role within CM2. Furthermore, we used an mRNA injection assay (Novak et al., 2016) to compare the localization of WT GFP-Cnn, GFP-Cnn-ΔCM2, and GFP-Cnn-HCAA in *cnn* mutant embryos expressing Spd-2-RFP as a centrosomal marker—as Cnn is not required to recruit Spd-2 to centrosomes in embryos (Conduit et al., 2014b). The HCAA mutation perturbed Cnn-scaffold assembly in vivo, although not to the same extent as deleting the entire CM2 domain (Figure S2B), suggesting that Zn^2+^ binding enhances scaffold assembly in vivo but is not essential. We suspect that this is because although Zn^2+^ is essential for the dimerization of CM2 in vitro, it is probably not essential for the dimerization of full-length Cnn molecules in vivo.

**Figure S3:**
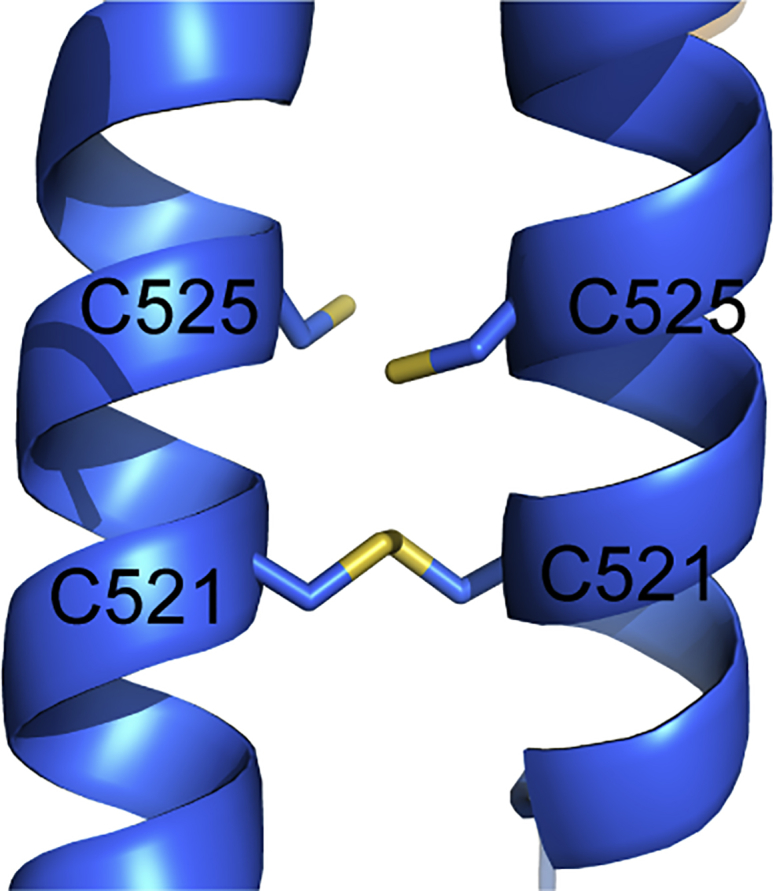
The LZ Domain Contains Cys Residues that Form Disulphide Bonds in the Crystal Structure, Related to Figure 2 A side-on view showing the Cys residues that form disulphide bonds in the LZ portion of the LZ:CM2 structure.

Two Cys residues conserved within *Drosophila* species in the LZ region (C521, C525) line the interior of the coiled coil, with C521 consistently forming a homotypic disulfide bond (Figure S3). Although the cytoplasm is generally a reducing environment, redox-controlled regulation events have been reported at the centrosome (Lim et al., 2015). However, mutation of both Cys residues to Ser had no obvious effect on Cnn-scaffold assembly in vivo (data not shown), suggesting that disulfide formation within the LZ is not essential for function under normal conditions.

### Perturbing the LZ:CM2 Interaction In Vitro Perturbs Cnn-Scaffold Assembly In Vivo

**Figure S4:**
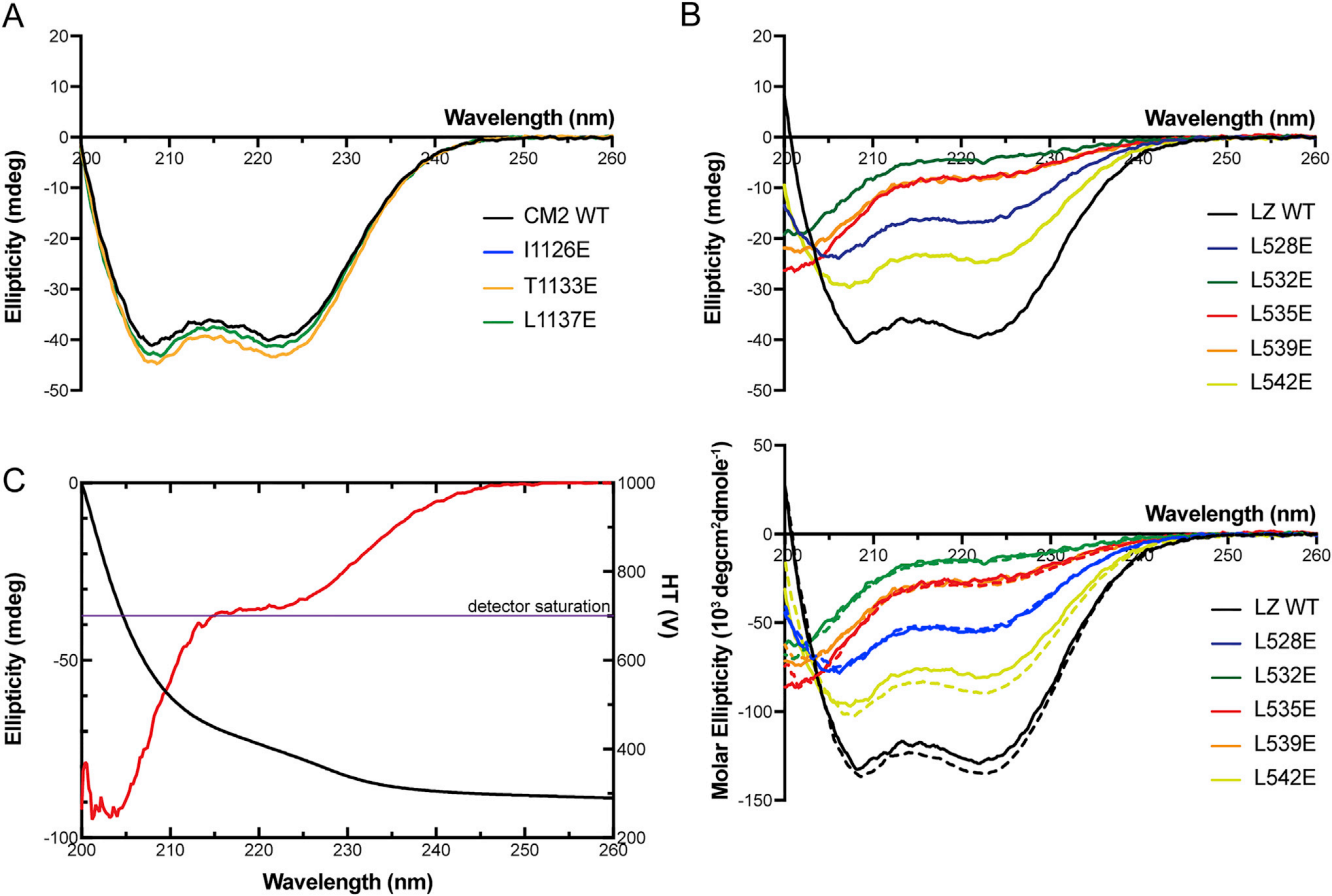
A Circular Dichroism Analysis of WT and Mutant CM2 and LZ Proteins, Related to Figure 3 (A) Circular Dichroism (CD) analysis showing that the WT-CM2 and various mutant-CM2 proteins are all largely helical in nature. (B) CD analysis showing that the WT-LZ protein is largely helical in nature, but the helical nature of the various mutant-LZ proteins is disrupted to varying degrees. The top traces show a single analysis for each protein at 0.2 mg/ml, while the bottom traces compare the analysis for each protein at 0.2 mg/ml (solid lines) and 0.6 mg/ml (dotted lines). This analysis reveals that none of the proteins show a tendency to become more helical at higher concentrations. This is important, as the helicity of the L535E mutant (*red* line) is strongly disrupted, yet the crystal structure reveals that this protein is largely helical when bound to CM2 (Figures 4D and 4E); this strongly suggests that binding to CM2 can induce the proper folding of the LZ domain, a result that supports the idea that CM2 can bind to and stabilize a partially unwound PReM domain (Figure 6). (C) CD analysis of LZ-L535E at 0.8 mg/ml showing the ellipticity (*red* line) and the HT voltage (*black* line). Also marked is a line representing the HT voltage threshold for reliable signal (700 V). At this concentration the detector signal becomes unreliable at 205 nm, close to the first negative for α-helical signal at 208 nm.

**Figure S5:**
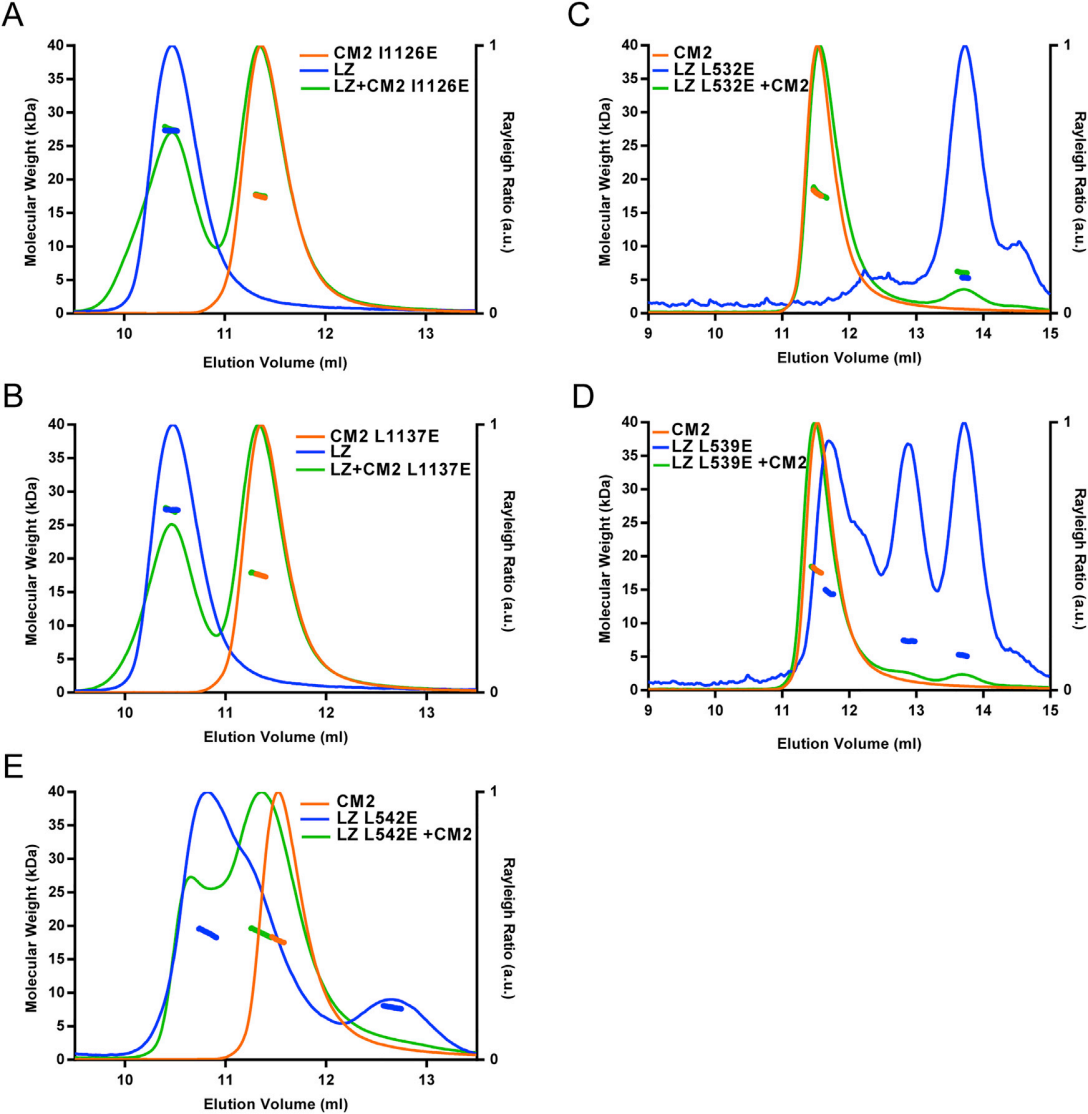
SEC-MALS Analysis of Various Mutant LZ or Mutant CM2 Proteins and Mutant LZ:CM2 Complexes, Related to Figure 3 (A–E) SEC-MALS analyses of the additional LZ and CM2 mutants whose behavior is summarized, but not shown, in Figure 3. CM2-I1126E (A), CM2-L1137E (B), LZ-L532E (C), LZ-L539E (D), LZ-L542E (E).

To test whether the LZ:CM2 interaction was required for Cnn-scaffold assembly in vivo, we substituted several individual residues that would be predicted to disrupt the interaction interface within the four-helix bundle (Figure 4A). We first concentrated on the three invariant residues in the CM2 domain (Ile1126, Thr1133, and Leu1137) (Figure 4A, orange residues). We individually substituted each residue with the large negatively charged residue Glu and confirmed that each substitution abolished the LZ:CM2 interaction in vitro; importantly, none of these substitutions appeared to perturb the ability of CM2 to adopt a helical conformation (Figure S4A) or to dimerize (Figures 3B, 3E, S5A, and S5B). We injected mRNAs encoding either the WT or the individually substituted GFP-Cnn fusion proteins into *cnn* mutant embryos expressing Spd-2-RFP. Strikingly, all of the individual substitutions perturbed Cnn localization and scaffold assembly, essentially to the same extent as deleting the entire CM2 domain (Figure 4C).

We also substituted the R1141 residue of the CM2 domain. This residue is highly conserved (Figuress 2A and S1A, light green asterisk), but it lies just outside the structured LZ:CM2 interaction interface, in the C-terminal 8aa that are largely disordered. R1141 is required for CM2 to interact with two other proteins, PLP and Centrocortin (Kao and Megraw, 2009, Lerit et al., 2015). Substituting R1141 for His abolishes these interactions, but this substitution had only a minor effect on Cnn localization and scaffold assembly compared to the CM2 deletion (Figure 4C)—although the Cnn flares at the periphery of the PCM were destabilized due to the failure to interact with PLP, as shown previously (Lerit et al., 2015, Richens et al., 2015). Thus, the previously described interactions between CM2 and PLP or Centrocortin are not sufficient to explain the role of CM2 in centrosomal targeting or scaffold assembly.

We next individually substituted each of the Leu residues in the LZ involved in the interaction with CM2 (L528, L532, L535, L539, L542) (Figure 4A, blue residues) for Glu. Most of these substitutions strongly perturbed the ability of the LZ to form a stable 2:2 tetramer with CM2 (Figures 3C, 3F, and S5C–S5E), the only exception being L535E, which still formed a 2:2 tetramer but with reduced efficiency (Figure 3D). Intriguingly, all of the individual LZ substitutions, including L535E, perturbed the assembly of the apo-LZ-homo-tetramer (Figures 3C, 3D, and S5C–S5E), and strongly reduced the α helicity of the proteins (Figures S4B and S4C).

We injected mRNAs encoding either WT-, complete LZ-deletion-, or individual LZ-substitution-GFP-Cnn fusion proteins into *cnn* mutant embryos expressing Spd-2-RFP. Unlike GFP-Cnn-ΔCM2, GFP-Cnn-ΔLZ was still recruited around the mother centrioles, but, like GFP-Cnn-ΔCM2, it failed to assemble into a scaffold structure, as shown previously (Conduit et al., 2014a) (Figure 4B). Most of the individual LZ substitutions perturbed Cnn-scaffold assembly to a similar extent as deleting the LZ domain (Figures 3F and 4B). Most strikingly, however, the L535E substitution could still form a detectable, although less robust, Cnn scaffold (Figure 4B); this is in excellent agreement with our analysis of the behavior of the LZ-L535E:CM2 complex in vitro (Figure 3D). Taken together, these mutational studies provide strong evidence that the LZ:CM2 interaction is important for Cnn-scaffold assembly.

### LZ-L535E Is Partially Unfolded but Can Be Induced to Fold by CM2

The purified LZ-L535E protein exhibits reduced α helicity, suggesting that it is partially unfolded (Figures S4B and S4C, red line), yet it can still interact with CM2 (Figure 3D). To better understand why the L535E substitution did not more strongly disrupt the LZ:CM2 interaction, we generated LZ-L535E:CM2 crystals and solved their structure (Figures 4D and 4E; Table S1). Perhaps surprisingly, LZ-L535E appeared to be folded normally and was largely helical within the crystal structure. Although L535 is packed within the core of the LZ:CM2 assembly, there is sufficient space in the core to accommodate the longer E535 side chain, and E535 also forms a hydrogen bond with the invariant CM2 T1133 residue in the core. This presumably explains why the L535E mutation only relatively mildly perturbs LZ:CM2 assembly in vitro (Figure 3D) and Cnn scaffold assembly in vivo (Figure 4B). Moreover, these data suggest that the interaction with CM2 is sufficient to order a partially disordered LZ; this may be important for CM2 function in vivo (see Discussion).

### Additional Sequences Surrounding the LZ Domain Allow the LZ and CM2 Domains to Assemble into Micron-Scale Structures

**Figure 5:**
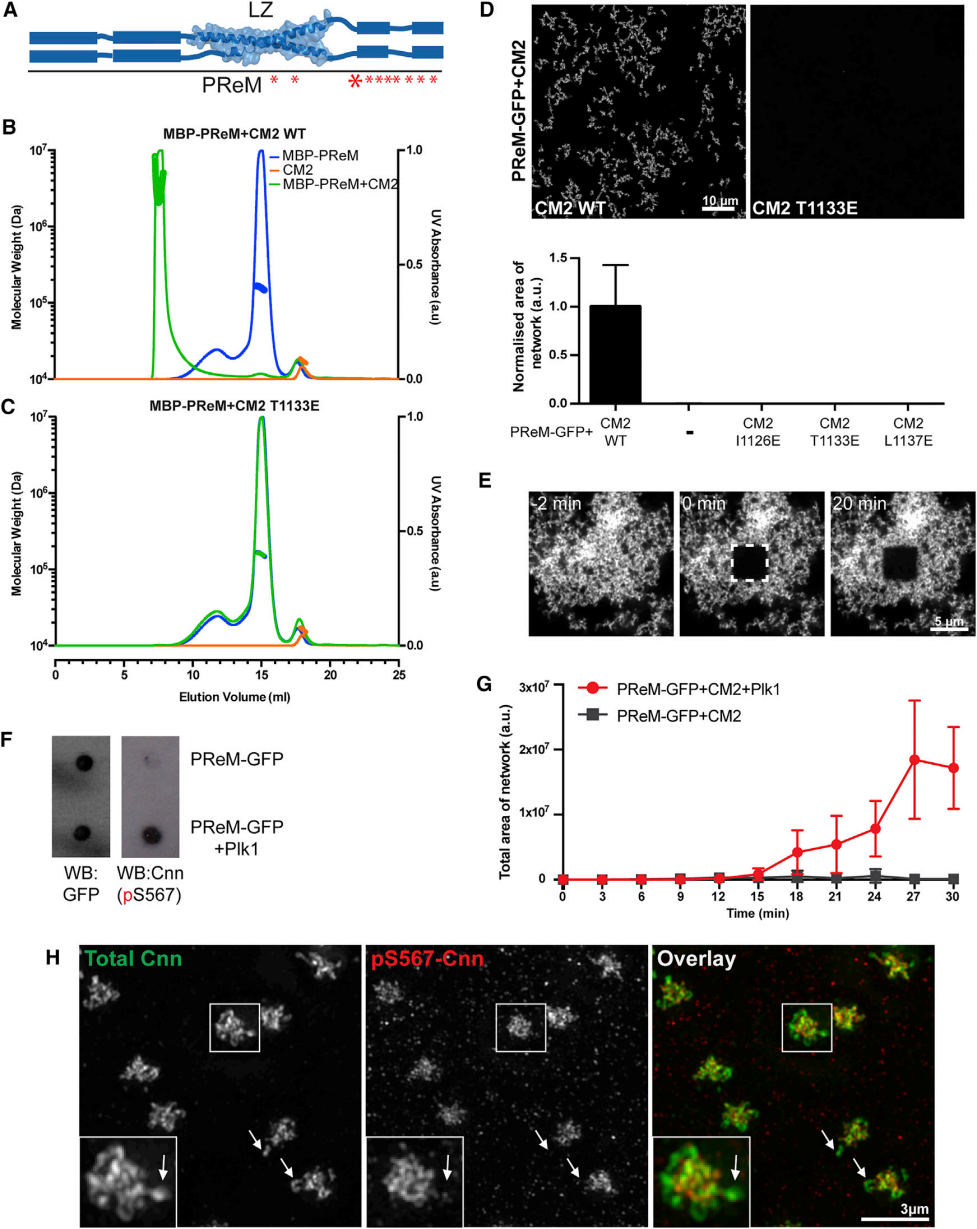
The PReM Domain Interacts with CM2 and Forms Large, Micron-Scale Complexes Whose Assembly Is Promoted by Plk1-Mediated Phosphorylation (A) Schematic illustration of the PReM domain showing the internal LZ (taken from the LZ:CM2 crystal structure) and surrounding sequences that are predicted to be helical (Jones, 1999) (blue bars). The ten Ser/Thr residues potentially phosphorylated by Polo are indicated by red asterisks; the larger asterisk represents the S567 residue used to raise the phospho-specific antibody. (B and C) SEC-MALS analysis of MBP-PReM (blue) and either CM2 (B) or CM2-T1133E (C) (orange) and MBP-PReM+CM2 (B) or MBP-PReM+CM2-T1133E (C) (green). (D) Chart quantifies and micrographs show examples of the micron-scale complexes visible by fluorescence microscopy when PReM-GFP is mixed with either WT (left) or mutant (example shown T1133E, right) forms of CM2. Error bars indicate SD. Both proteins are at a final concentration of 20 μM. (E) Micrographs show a FRAP analysis of protein turnover in the PReM-GFP:CM2 complexes (as seen in [D]). Complexes were imaged (t = −2 min), a small area was photobleached (t = 0 min), and fluorescence recovery monitored (t = 20 min). (F) Western dot-blot shows that PReM-GFP is phosphorylated by purified Plk1 in vitro, allowing the phospho-specific Cnn-pS567 antibody to recognize the protein; the same blot was probed with anti-GFP antibodies to confirm equal loading of PReM-GFP. (G) Graph quantifies the visible area of the PReM-GFP:CM2 complexes (both proteins at a final concentration of 10 μM) at various time points after mixing when the PReM-GFP protein has been pre-treated with Plk1 (red line) or with buffer control (gray line). Error bars indicate SD (n = three independent experimental replicates; note that one outlier time point in one experiment that was ∼10 times brighter than all the others was excluded from this analysis). (H) Micrographs show how anti-Cnn-p567 antibodies (red) preferentially recognize the inner region of the centrosome and are largely absent from the more peripheral regions (in some cases highlighted with arrows) recognized by antibodies that recognize total Cnn (green). See also Figure S6. Scale bars = 10 μm (A), 5 μm (E), and 3 μm (H).

**Figure S6:**
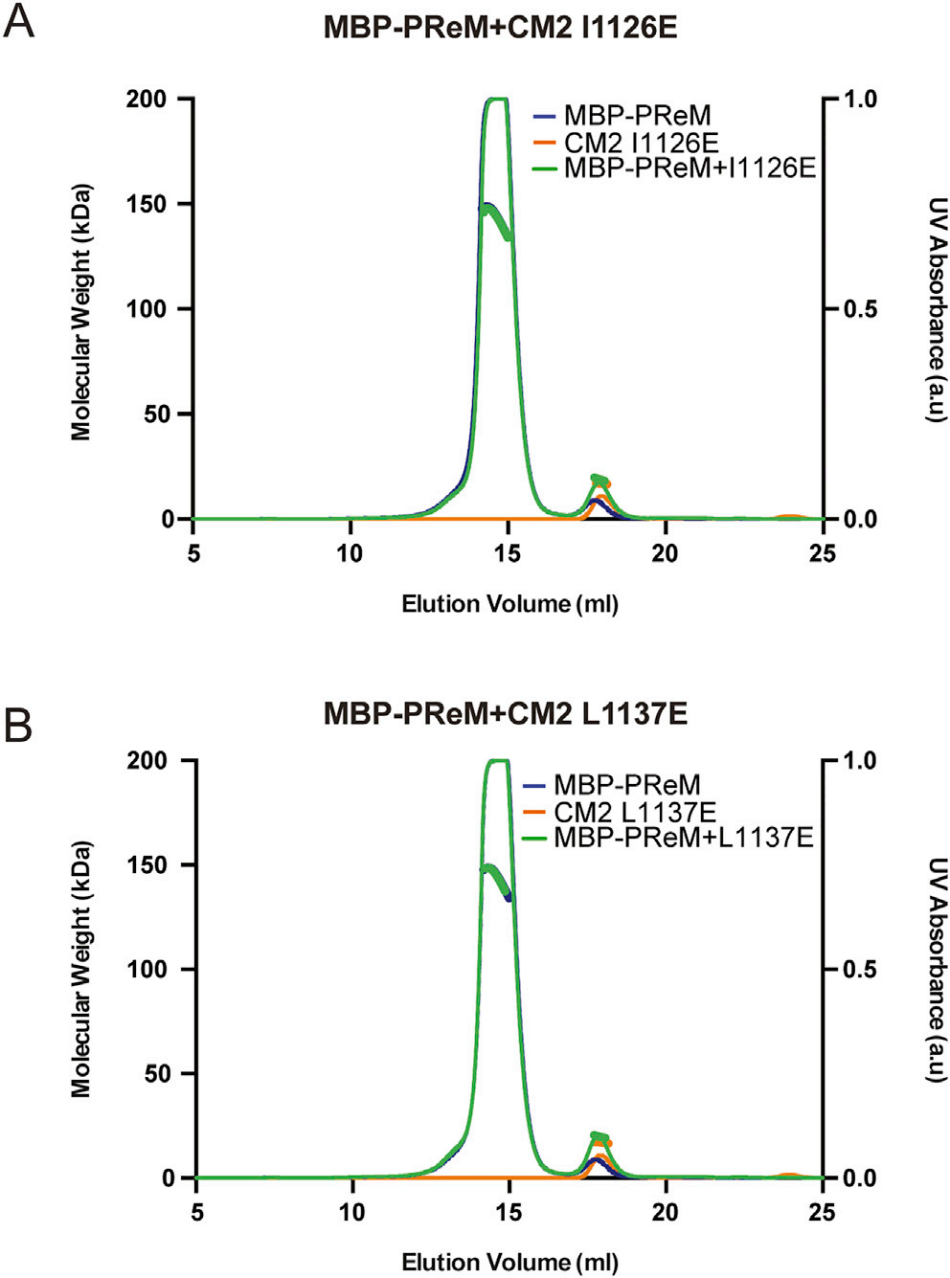
SEC-MALS Analysis of PReM-Domain Binding to Mutant CM2 Proteins, Related to Figure 5 (A and B) SEC-MALS analyses showing the inability of the CM2 mutants CM2-I1126E (A) and CM2-L1137E (B) to bind MBP-PReM.

The originally defined PReM domain (aa403-608) comprises the LZ (aa490-544) and additional C-terminal (aa545-608) and N-terminal (aa403-489) extensions that are predicted to be largely helical (Figure 5A, blue bars). The C-terminal extension also contains multiple Ser/Thr residues that are phosphorylated by Plk1 in vitro and that promote Cnn-scaffold assembly in vivo (Conduit et al., 2014a) (Figure 5A, red asterisks). We purified an MBP-PReM fusion, and, as shown previously (Conduit et al., 2014a), it behaved as a dimer (Figure 5B). When mixed with CM2, however, MBP-PReM assembled into complexes that were much larger than the LZ:CM2 tetramer (Figure 5B). Importantly, the CM2 I1126E, T1133E, and L1137E mutations all completely abolished the formation of these larger complexes (Figures 5C and S6), strongly arguing that they are not simply nonspecific aggregates but are mediated by the same molecular interactions seen in our hetero-tetrameric LZ:CM2 complex.

Previous studies have shown that purified full-length *C. elegans* SPD-5 can assemble into micron-scale complexes in vitro (Woodruff et al., 2015, Wueseke et al., 2016), and we wondered whether the PReM:CM2 complexes might have a similar ability. A purified PReM-GFP fusion protein did not form visible complexes, but when mixed with CM2, it assembled into large micron-scale structures that resembled those formed by purified SPD-5 (Figure 5D). A FRAP analysis revealed that the molecules in these structures exhibited no detectable tendency to internally re-arrange, strongly suggesting that they are solid-like rather than gel- or liquid-like structures (Figure 5E) (see Discussion).

Importantly, the assembly of the micron-scale PReM-GFP:CM2 structures was also abolished by any one of the CM2 mutations that disrupted LZ:CM2 complex assembly (I1126E, T1133E, and L1137E), again arguing that these structures are not simply nonspecific aggregates (Figure 5D).

### Phosphorylation by Plk1 Promotes the Assembly of the PReM-GFP:CM2 Structures In Vitro

A key feature of the assembly of the Cnn scaffold in vivo is that it appears to be initiated at centrosomes by the Polo-dependent phosphorylation of Cnn at multiple sites within the PReM domain (Conduit et al., 2014a). To test whether the assembly of the PReM-GFP:CM2 structures in vitro was influenced by Plk1-dependent phosphorylation, we first raised an antibody that specifically recognized a PReM-domain peptide that had been phosphorylated on one of the conserved Ser residues (S567) (Figure 5A, large red asterisk,). Western blotting revealed that this antibody only recognized PReM-GFP after it had been phosphorylated by Plk1 in vitro, confirming that the antibody was phospho-specific and that Plk1 can phosphorylate PReM-GFP in vitro, as shown previously for MBP-PReM (Conduit et al., 2014a) (Figure 5F). Strikingly, pre-incubation of the PReM domain with Plk1 dramatically increased the efficiency of PReM-GFP:CM2 complex assembly (Figure 5G). Thus, the co-assembly of these two domains into micron-scale assemblies in vitro appears to be regulated by phosphorylation in the same way that Cnn assembly into a centrosomal scaffold is regulated in vivo.

### Cnn Is Preferentially Phosphorylated in the Inner Region of the PCM

We have previously speculated that Cnn-scaffold assembly is initiated by Polo-dependent phosphorylation around the mother centriole, while disassembly is initiated toward the centrosomal periphery because Cnn molecules are gradually dephosphorylated as they flux outward away from the source of Polo around the mother centriole (Conduit et al., 2014a, Conduit et al., 2015). The Cnn-phospho-S567 antibody preferentially recognizes Cnn phosphorylated by Plk1 (Figure 5F), allowing us to directly test this possibility. In support of our hypothesis, we found that the Cnn-phospho-S567 antibody preferentially recognized the inner region of the Cnn scaffold and was largely absent from the peripheral regions where the Cnn scaffold was starting to disassemble (Figure 5H, arrows).

## Discussion

**Figure 6:**
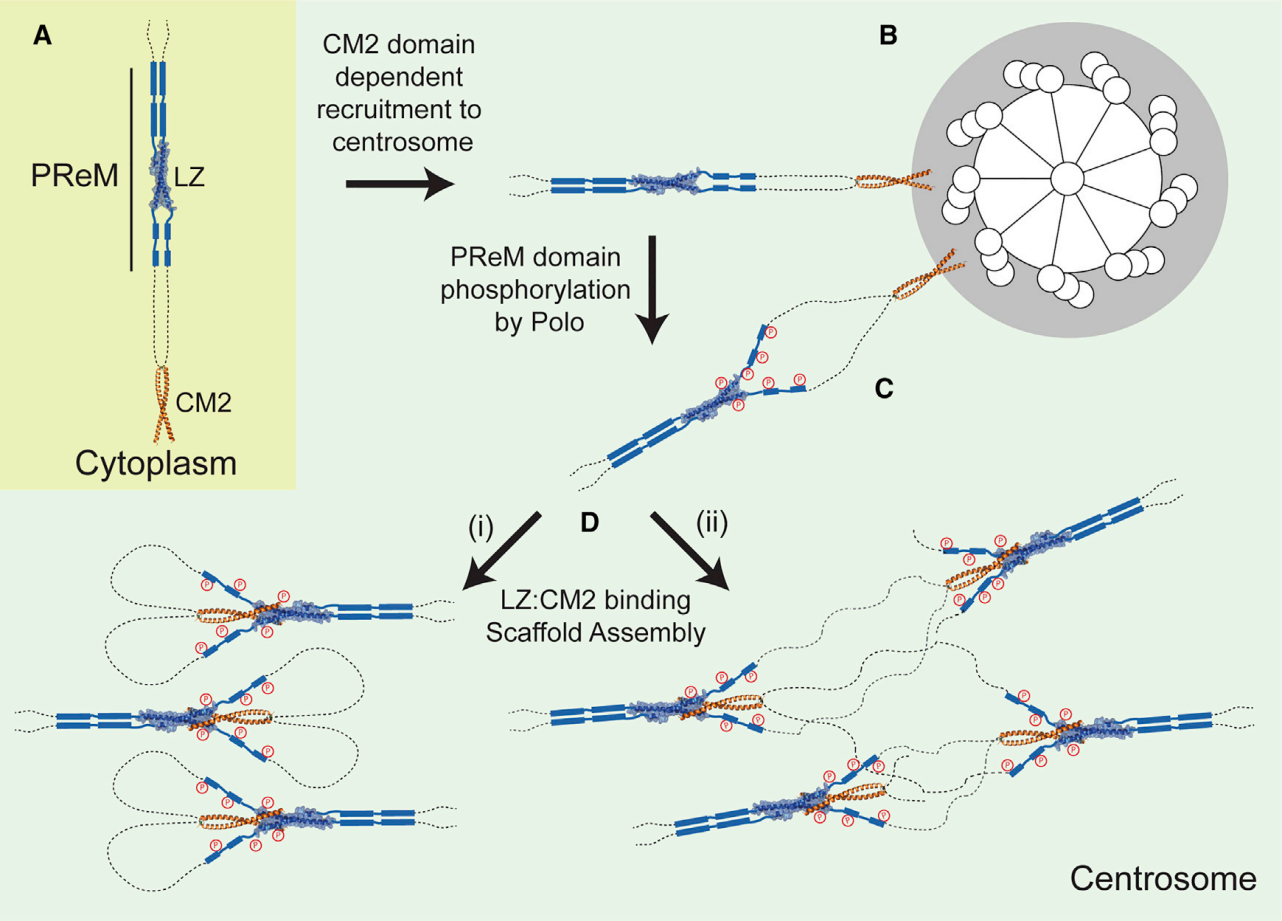
A Schematic Illustration of How Cnn Molecules Might Assemble into a Scaffold around the Mother Centriole (A) In the cytoplasm, Cnn exists as a dimer: the PReM-LZ and CM2 structures are highlighted, and the other sequences within Cnn are depicted with dotted lines (not to scale). (B) Cnn dimers are recruited to the mother centriole through their CM2 domains. (C) These molecules are phosphorylated in their PReM domains—and almost certainly at several other sites that are not depicted here (Conduit et al., 2014a). Phosphorylation destabilizes the helical dimer, allowing it to partially “unwind.” (D) The partial unwinding of the dimer allows the CM2 domain to interact with the LZ, either intra-molecularly (i) or inter-molecularly (ii); the partial unwinding of the helices could allow the Cnn molecules, which are predicted to consist largely of coiled-coil domains but also contain predicted disordered regions (Figure 2A), extra flexibility to form an intra-molecular interaction. The formation of the LZ:CM2 complex allows Cnn molecules to assemble into larger complexes; two models of how this might occur are shown here. Note that in (i), phosphorylation destabilizes *intra*-molecular PReM domain interactions but does not prevent *inter*-molecular PReM domain interactions. We think this plausible, as the intra-molecular dimer might initially be slightly destabilized by phosphorylation but then more strongly destabilized by the binding of CM2, thus favoring inter-molecular interactions. An alternative possibility is that phosphorylated PReM domains no longer tend to form dimers but tend to form higher-order oligomers (although this is not illustrated here). There is some evidence for this idea as, in vitro, MBP-PReM forms a stable dimer, whereas MBP-PReM-10D/E forms larger oligomers (Conduit et al., 2014a).

Cnn plays a crucial role in mitotic centrosome assembly in *Drosophila* (Lucas and Raff, 2007, Megraw et al., 1999, Megraw et al., 2001, Vaizel-Ohayon and Schejter, 1999). We have identified two conserved regions of *Drosophila* Cnn—an internal leucine zipper (LZ) and the C-terminal Cnn-motif-2 (CM2)—that are important for this process. Structural analyses revealed that the LZ and CM2 domains form an antiparallel 2:2 complex of two parallel coiled coils. Mutagenesis confirmed that amino acids within the interaction interface that are required for LZ:CM2 complex assembly in vitro are also required for centrosome assembly in vivo. The LZ is contained within the previously identified phospho-regulated-multimerization (PReM) domain, which appears to be phosphorylated by Polo at centrosomes to drive the mitotic assembly of the Cnn scaffold in vivo (Conduit et al., 2014a). The PReM and CM2 domains can co-assemble into micron-scale structures in vitro, and this is enhanced by Plk1 phosphorylation of the PReM domain. These studies provide a first atomic insight into a structural interaction required to assemble the mitotic PCM. In Figure 6, we schematically illustrate how Cnn molecules might assemble into a scaffold and how this process might be regulated by phosphorylation so that it only occurs at centrosomes.

Cnn molecules are predicted to be largely coiled coils, so we propose that Cnn exists as a “closed” homo-dimer in the cytoplasm and that the LZ and CM2 domains do not interact within these dimers (Figure 6A). Cnn dimers are recruited around the mother centriole (Figure 6B) where, during mitosis, they can be phosphorylated by Polo (Figure 6C) (Conduit et al., 2014a), which is highly concentrated around the mother centriole (Conduit et al., 2015, Fu and Glover, 2012). We speculate that phosphorylation destabilizes the PReM domain helical dimer, allowing the helices to partially unwind (Figures 6B → 6C). This allows the C-terminal CM2 to “invade” the partially unwound dimer—either intra-molecularly (Figure 6Di) or inter-molecularly (Figure 6Dii)—to form a tight LZ:CM2 complex. The LZ:CM2 interaction stabilizes the partially unwound state of the nearby helices, allowing them to form inter-molecular interactions with other unwound helical elements in nearby Cnn molecules, assembling a matrix-like structure. In fly embryos, the Cnn scaffold fluxes away from the mother centriole, separating Cnn molecules from the source of Polo kinase. As a result, Cnn is gradually dephosphorylated, favoring the re-establishment of the intra-molecular dimer interactions within the PReM domain, and so scaffold disassembly.

These ideas can explain why Cnn-scaffold assembly normally only occurs at centrosomes and only during mitosis. Cnn must be phosphorylated at multiple sites to drive scaffold assembly (Conduit and Raff, 2010), and it seems likely that the mother centriole is the only place in the cell where the local concentration of both Cnn and Polo are high enough for this to occur when Polo becomes activated during mitosis. Our in vitro data support this hypothesis, as phosphorylation by Plk1 enhances the ability of the purified PReM and CM2 domains to co-assemble into micron-scale structures, suggesting that this key interaction is strongly influenced by Polo/Plk1-dependent phosphorylation. Within the context of the full-length Cnn molecule, the PReM and CM2 domains also do not appear to interact efficiently until Cnn has been phosphorylated—phospho-mimicking GFP-Cnn-10D/E molecules can efficiently form CM2-dependent scaffolds in the cytoplasm, while WT GFP-Cnn molecules cannot (Conduit et al., 2014a).

An important conclusion from our studies is that the key CM2 residues required for scaffold assembly are also required to recruit Cnn to centrosomes. This dual role for CM2 implies that Cnn molecules can only assemble into a scaffold once their CM2 domains have been released from their centrosomal recruiting sites (Figure 6C → 6D). A priori, this might seem surprising, but in these early embryos the Cnn scaffold continually assembles close to the mother centrioles and then fluxes outward along the centrosomal MTs (Conduit and Raff, 2015, Conduit et al., 2014a). Thus, the assembling Cnn scaffold appears to be released from its centrosomal recruiting sites to allow it to flux outward; this could be enforced by the dual role of CM2. An attractive possibility is that Polo phosphorylation at the centrosome not only “opens” the PReM domain to allow CM2 binding but also stimulates the release of CM2 from its centrosomal recruiting sites.

The nature of the PReM domain interactions that promote matrix assembly are presently unclear, but they could occur in several ways, two of which are depicted in Figure 6D. Coiled-coil-containing proteins have been implicated in a wide variety of cellular function, but, with the exception of de novo synthesized coils such as those that form hydrogels (Banwell et al., 2009), it is rare that they have been proposed to form large network structures. It is therefore interesting that a close structural homolog of the LZ:CM2 hetero-tetramer is the homo-tetrameric Homer1 protein that is essential for the construction of the mesh-like matrix structure of the cytoplasmic postsynaptic density (PSD) in neurons (Hayashi et al., 2009). Homer1 performs this function by combining a homotypic coiled-coil interaction at its C terminus with interactions with another multimeric protein (Shank) at its N terminus. The PReM and CM2 domains form a structurally analogous tetrameric coiled-coil bundle but can use interactions between additional elements within the PReM domain itself to assemble large multimeric structures without the need for further accessory proteins.

The mitotic centrosome is a non-membrane-bound organelle, and there has been much interest recently in the idea that such organelles may be formed by the phase-separation of components into “biomolecular condensates,” often with liquid- or gel-like properties (Banani et al., 2017). This is an attractive idea for the centrosome, as a PCM with liquid-like properties would allow the hundreds of proteins within the mitotic centrosome to interact efficiently (Zwicker et al., 2014; Hyman et al., 2014). We show here, however, that the PReM:CM2 proteins can effectively phase separate from solution to form what appears to be a solid-phase—as the molecules within these structures do not detectably internally rearrange over a timescale of several minutes (Figure 5E). Moreover, dynamic studies suggest that Cnn molecules also do not readily internally re-arrange at centrosomes in vivo (Conduit et al., 2014a), and this also appears to be the case for SPD-5 in worm embryos (Laos et al., 2015).

Interestingly, however, purified SPD-5 can also phase separate from solution, but it appears to form either a solid- (Woodruff et al., 2015) or gel-like phase (Woodruff et al., 2017), depending on the conditions—although the gel-like phase seems to rapidly “mature” into a form where the SPD-5 molecules do not readily undergo internal rearrangements (Woodruff et al., 2017). Thus, we do not rule out the possibility that full-length Cnn molecules could also form a gel-like phase under certain conditions. To date, most proteins that form condensates with liquid-like properties do so using intrinsically disordered domains or multiple low-affinity binding sites (Banani et al., 2017), whereas the high-affinity, well-ordered, LZ:CM2 interaction we identify here is clearly central to scaffold assembly both in vitro and in vivo. Perhaps within the context of the full-length Cnn molecules the LZ:CM2 interaction serves as an internal scaffold that allows other regions of Cnn to form other, more disordered and/or multivalent-low-affinity interactions that allow Cnn molecules to form a gel-like phase. Our finding that two such small regions of Cnn can self-assemble into micron-scale structures in vitro provides a powerful tool with which to address this important question.

## STAR★Methods

### Key Resource Table

**Table:** 

REAGENT or RESOURCE	SOURCE	IDENTIFIER
**Antibodies**		

Mouse monoclonal anti-GFP (clones 7.1 and 13.1)	Roche	Cat#11814460001; RRID: AB_390913
Mouse monoclonal anti-Actin (clone AC-40)	Sigma-Aldrich	Cat#A3853; RRID: AB_262137
Rabbit phospho antibody anti- Cnn pSer567	Pocono Rabbit Farm & Laboratory	Animal#30129; Lab ID: Ab#236
Sheep polyclonal anti-Cnn	Cottee et al., 2013. Raised in-house	Lab ID: Ab#219
Anti-Sheep IgG Alexa Fluor 488	Invitrogen, Thermo Fisher Scientific	Cat#A11015; RRID: AB_141362
Anti-Rabbit IgG Alexa Fluor 594	Invitrogen, Thermo Fisher Scientific	Cat#A11037; RRID: AB_2534095
Amersham ECL Mouse IgG, HRP-linked whole antibody (from sheep)	GE Healthcare Life Sciences	Cat#NA931V; RRID: AB_772210
Amersham ECL Rabbit IgG, HRP-linked whole antibody (from donkey)	GE Healthcare Life Sciences	Cat#NA934V; RRID: AB_772206

**Chemicals, Peptides, and Recombinant Proteins**		

Red fluorescent polystyrene beads	Thermo Fisher	Cat#F8808
TetraSpeck beads	Thermo Fisher Scientific; Micron Oxford	Cat#T14792
Adeosine 5′-triphosphate disodium salt hydrate (ATP)	Sigma-Aldrich	Cat#A7699
β-Glycerophosphate disodium salt hydrate	Sigma-Aldrich	Cat#G9422
Roche complete	Roche	Cat#11873580001
QuikChange II XL Site-Directed Mutagenesis kit	Agilent	Cat#200522
Ambion, mMESSAGE mMACHINE T3 Transcription kit	Termo Fisher	Cat#AM1348
Gateway LR Clonase Enzyme mix	Thermo Fisher	Cat#11791019
Recombinant protein: Cnn LZ WT (GPM-D490-K544/L552/S567)	This paper	N/A
Recombinant protein: Cnn CM2 WT (GGS-H1082-L1148)	This paper	N/A
Recombinant protein: Cnn CM2 WT (GGS-H082-L1148-EFGENLYFQ)	This paper	N/A
Recombinant protein: Cnn PReM (M-Q403-H608)	This paper	N/A
Recombinant protein: GST-3C protease	Made in house	N/A
Recombinant protein: TEV protease	Made in house	N/A

**Critical Commercial Assays**		

PLK1 in vitro kinase assay	ProQinase	Cat#0183-000-1

**Deposited Data**		

LZ (aa490-544) -CM2 complex structure	This paper	PDB: 5MVW
LZ L535E-CM2 complex structure (Crystal Form P2_1_)	This paper	PDB: 5MW0
LZ L535E-CM2 complex structure (Crystal Form C2)	This paper	PDB: 5MW9
LZ (aa490-567)-CM2 complex structure	This paper	PDB: 5MWE
CM2 apo domain structure	This paper	PDB: 5I7C

**Experimental Models: Organisms/Strains**		

*E. coli* B834 (DE3)	Novagen	Cat#69041
*E. coli* C41 (DE3)	Lucigen	Cat#60442
w67 (as wild type)	Lab stock	N/A
pUb-RFP-Spd2, cnn^f04547^/ SM6ˆTM6	Conduit et al., 2014a	Lab ID: Paul’s F060
cnn^HK21^/SM6ˆTM6	Based on Megraw et al., 1999	Lab ID: F397
w;pUbq-GFP Cnn WT, cnn^f04547^/ SM6ˆTM6	Lucas and Raff, 2007	Lab ID: T054
w;pUbq-GFP Cnn-ΔCM2, cnn^f04547^/SM6a	This paper	Lab ID: ZF1-9, box III

**Oligonucleotides**		

Primer: ΔLZ_F: GTGCTCTTCCAGCGCCTGGCAGACCACAAAGATGTTCTTGGCGTGTTG	This paper	N/A
Primer: ΔLZ_R: CAACACGCCAAGAACATCTTTGTGGTCTGCCAGGCGCTGGAAGAGCAC	This paper	N/A
Primer: attBCnn1_F: GGGGACAAGTTTGTACAAAAAAGCAGGCTTCATGGACCAGTCTAAACAG GTTTTG	This paper	N/A
Primer: attBCnn1081_R GGGGACCACTTTGTACAAGAAAGCTGGGTCCTATACTGTGGCTGCACCAGTTG	This paper	N/A
Primers for LZ constructs, see Table S2	This paper	N/A
Primers for CM2 constructs, see Table S2	This paper	N/A
Primer: Cnn403_F: CTTTAAGAAGGAGACTCGAGATGCAGTTGCAGACGGAAGTAAAGAAG	This paper	N/A
Primer: Cnn608_F: GGTTTTCGGATCCAGGTTCGTGGCTTGCATCACCTTCG	This paper	N/A

**Recombinant DNA**		

pRNA GFP Cnn	This paper	N/A
pRNA GFP Cnn 10D/E	Conduit et al., 2014a	N/A
pRNA GFP Cnn-ΔLZ (aa490-544)	This paper	N/A
pRNA GFP Cnn L528E	This paper	N/A
pRNA GFP Cnn L532E	This paper	N/A
pRNA GFP Cnn L535E	This paper	N/A
pRNA GFP Cnn L539E	This paper	N/A
pRNA GFP Cnn L542E	This paper	N/A
pRNA GFP Cnn-ΔCM2 (aa1082-1148)	This paper	N/A
pRNA GFP Cnn HCEE	This paper	N/A
pRNA GFP Cnn I1126E	This paper	N/A
pRNA GFP Cnn T1133E	This paper	N/A
pRNA GFP Cnn L1137E	This paper	N/A
pRNA GFP Cnn R1141H	This paper	N/A
pRNA GFP Cnn 10D/E-ΔCM2	This paper	N/A
pETM44 His_6_MBP-LZ (aa490-544/552/567)	This paper	N/A
pETM44 His_6_MBP-LZ (aa490-544 L528E)	This paper	N/A
pETM44 His_6_MBP-LZ (aa490-544 L532E)	This paper	N/A
pETM44 His_6_MBP-LZ (aa490-544 L535E)	This paper	N/A
pETM44 His_6_MBP-LZ (aa490-544 L539E)	This paper	N/A
pETM44 His_6_MBP-LZ (aa490-544 L542E)	This paper	N/A
pLip CM2 (aa1082-1148)	This paper	N/A
pLip CM2 (aa1082-1148 HCAA)	This paper	N/A
pLip CM2 (aa1082-1148 I1126E)	This paper	N/A
pLip CM2 (aa1082-1148 T1133E)	This paper	N/A
pLip CM2 (aa1082-1148 L1137E)	This paper	N/A
pWaldo PReM (aa403-608)-GFP-A206K	This paper	N/A

**Software and Algorithms**		

ImageJ	NIH	Version 2.0.0
Prism	GraphPad	Version 7
Xia2 pipeline	Winter, 2010	www.ccp4.ac.uk
AIMLESS	Evans and Murshudov, 2013	www.ccp4.ac.uk
Phenix AutoSol	Adams et al., 2010	www.phenix-online.org
Phenix Autobuild	Terwilliger et al., 2013	www.phenix-online.org
Phenix.refine	Adams et al., 2010	www.phenix-online.org
Phaser	McCoy et al., 2007	www.phenix-online.org
Coot	Emsley and Cowtan, 2004	www2.mrc-lmb.cam.ac.uk/personal/pemsley/coot/
Phenix ReadySet	Adams et al., 2010	www.phenix-online.org
AutoPROC	Vonrhein et al., 2011	www.globalphasing.com
TLSMD server	Painter and Merritt, 2006	skuld.bmsc.washington.edu/∼tlsmd/
Buccaneer	Cowtan 2006	www.ccp4.ac.uk
Refmac	Murshudov et al., 2011	www.ccp4.ac.uk
Molrep	Vagin and Teplyakov, 2010	javascript:void(0);
PyMOL	PyMOL	www.pymol.org
ASTRA 6.1.1.17 software	Wyatt	www.wyatt.com/products/software/astra.html
PSIPRED	Buchan et al., 2013	bioinf.cs.ucl.ac.uk/psipred/
COILS	Lupas et al., 1991	www.ch.embnet.org/software/COILS_form.html
XtalPred-RF	Slabinski et al., 2007	ffas.burnham.org/XtalPred-cgi/xtal.pl
ClustalX	Larkin et al., 2007	www.clustal.org/clustal2/
SOCKET	Walshaw and Woolfson, 2001	coiledcoils.chm.bris.ac.uk/socket/server.html
Volocity 6.3	PerkinElmer Inc.	cellularimaging.perkinelmer.com/
softWoRx 5.5.1	GE Healthcare Life Sciences	www.gelifesciences.com/
softWoRx 6.1	GE Healthcare Life Sciences	www.gelifesciences.com/

**Other**		

Perkin Elmer ERS Spinning Disk confocal system	PerkinElmer Inc.	No longer available
DeltaVision Elite microscope	GE Healthcare Life Sciences; Micron Oxford	www.gelifesciences.com/
DeltaVision OMX V3 Blaze microscope	GE Healthcare Life Sciences; Micron Oxford	Cat#29065721
Zeiss 880 Airy-scan microscope	Zeiss International; Micron Oxford	www.zeiss.com

### Contact for Reagent and Resource Sharing

Further information and requests for resources and reagents should be directed to and will be fulfilled by the Lead Contact, Jordan Raff (jordan.raff@path.ox.ac.uk).

### Experimental Model and Subject Details

#### Fly husbandry, stocks and handling

*Drosophila melanogaster w*^*67*^ flies (a wild-type line carrying a point mutation in the white gene) were used as a WT stock in all experiments and *yw* flies were used as the parental stock in the generation of transgenic lines. Balancer chromosomes and markers used have been described previously (Flybase, USA). Flies were kept at 25°C or 18°C on *Drosophila* culture medium (0.77% agar, 6.9% maize, 0.8% soya, 1.4% yeast, 6.9% malt, 1.9% molasses, 0.5% propionic acid, 0.03% ortho-phosphoric acid and 0.3% nipagin). Stocks were kept in 8 cm x 2.5 cm plastic vials or 0.25-pint plastic bottles. Embryos were collected on cranberry-raspberry juice plates (25% cranberry-raspberry juice, 2% sucrose and 1.8% agar) supplemented with fresh yeast. Standard fly handling techniques were employed (Roberts, 1998). In vivo studies were performed using 1.5-2 hr-old embryos (syncytial blastoderm stage). After 0-1 hr collections at 25°C, embryos were aged at 25°C for 1 hr. When injecting mRNA, embryos were collected for 30 min and aged for 1.5 hr after mRNA injection. Prior to injection or imaging, embryos were dechorionated by using double-sided tape onto a slide and mounted on a strip of glue onto a 35 mm glass bottom petri dish with a 14 mm micro-well (MatTek). After desiccation for 1 min at 25°C, embryos were covered in Voltalef oil (ARKEMA).

#### Organisms for in vitro studies

*Escherichia coli* cells were cultured in LB or TB medium (see Method details).

### Method Details

#### In vitro mRNA production and injection

The mRNA injection assay we use here is based on that described previously (Novak et al., 2014). Full length Cnn was PCR amplified from the cDNA (UniProt reference number: P54623-2) and sub-cloned into a modified pRNA destination vector (Conduit et al., 2014a) using the Gateway cloning system (Life Technologies). The vector encodes a T3 RNA polymerase promoter and an N-terminal GFP fusion tag. The deletion construct Cnn-ΔLZ, was generated using a Quikchange II XL mutagenesis kit (Agilent), by ‘looping out’ the region of interest (aa490-544) on pRNA-GFP-Cnn. Cnn-ΔCM2 was PCR amplified from the cDNA (UniProt reference number: P54623-2) and then sub-cloned into pRNA destination vector by the Gateway cloning system (Life Technologies). All the point mutations described in the text were introduced into the *cnn* coding sequence by Quikchange mutagenesis, using *Drosophila*-optimized codons for each substituted residue. PReM domain Phospho-mimetic constructs were generated as described (Conduit et al., 2014a). Primers used for cloning in this paper are listed in Table S2.

mRNA was synthesized in vitro using an mMESSAGE mMACHINE T3 Transcription Kit (Life Technologies) and purified using RNeasy MinElute kit (QIAGEN). The mRNA concentration was adjusted to 2 mg/ml and injected into fly embryos collected from *cnn*^*f04547*^*/cnn*^*HK21*^ hemizygous mutant mothers expressing a Spd-2-RFP fusion protein (Conduit et al., 2014a). The injected embryos were incubated at 25°C for 60-90 min to allow translation of the GFP-fusion protein. Embryos collected from fly lines and unfertilized eggs were collected similarly and incubated at 25°C for 60-90 min. Images were taken using a Perkin Elmer ERS spinning disk (Volocity software) mounted on a Zeiss Axiovert microscope using a 63X/1.4NA oil immersion objective and an Orca ER CCD camera (Hamamatsu Photonics, Japan).

#### Image analysis

We used ImageJ to calculate the average centrosomal fluorescence profile for the different Cnn mutants (both for fly lines and mRNA injected embryos), where 5 centrosomes/embryo were analyzed, in at least 5 separate embryos. The profile for an individual centrosome was calculated by finding the center of mass of the centrosome by thresholding the image and running the ‘‘analyze particles’’ (center of mass) macro on the most central Z plane of the centrosome as described (Conduit et al., 2014a). We then centered concentric rings (spaced at 0.021 μm and spanning across 4.18 μm) on this center and measured the average fluorescence around each ring (radial profiling). After subtracting the average cytosolic signal, each profile was normalized so the peak intensity of the WT GFP-Cnn was equal to 1, and other protein types normalized to this value. The profile was then mirrored to produce a full centrosome profile. The area under the curve was calculated using GraphPad Prism, and an average area for 5 centrosomes per embryo was calculated. At least 5 embryos were used to calculate the average area under the curve for each protein, and an unpaired t test was performed to assess the significance of any differences.

#### Transgenic *Drosophila* lines

All transgenic lines were generated by standard P-element mediated transformation (performed by the Fly Facility in the Department of Genetics University of Cambridge). The GFP-Cnn and GFP-ΔCM2 lines used were crossed into a *cnn* mutant background.

#### Western blot analysis

Western blotting to estimate embryonic protein levels was performed as described previously (Novak et al., 2014). The following primary antibodies were used for western blot analysis: mouse anti-GFP (1:500, Roche) and mouse anti-actin (1:1000, Sigma). Anti-mouse IgG, HRP-linked (1:3000, GE Healthcare) secondary antibody was used.

#### Recombinant protein expression and purification

##### The LZ Apo protein

The cDNA sequence encoding *Drosophila* Cnn_490-544_ (the LZ domain) was subcloned into a pETM44 (EMBL) vector encoding an N-terminal His_6_-MBP tag. Proteins were expressed in *Escherichia coli* (*E. coli*) B834 (DE3) strains in TB broth, and purified using Ni-NTA chromatography followed by size exclusion chromatography (50 mM Tris-HCl pH8.0, 150 mM NaCl, 5 mM β-mercaptoethanol). For SEC-MALS analysis and crystallization trials, the N-terminal MBP tag was cleaved off using GST-3C protease, dialyzed into 50 mM Tris-HCl pH7.5, 300 mM NaCl, 5 mM β-mercaptoethanol at 4°C overnight. The untagged protein was further purified via reverse Ni-NTA chromatography and size exclusion chromatography (50 mM Tris-HCl pH7.5, 150 mM NaCl, 5 mM β-mercaptoethanol).

##### The CM2 Apo protein

The cDNA sequence encoding *Drosophila* Cnn_1082-1148_ (the CM2 domain) was subcloned into a custom “pLip” vector (Cottee et al., 2013). This vector encodes a T7 polymerase promoter site and two, TEV protease cleavable, His-tagged lipoyl domains (from *Bacillus stearothermophilus dihydrolipoamide acetyltransferase*) that flank the insert. Note that a stop codon (TAA) was included at the 3′ end of DNA fragment encoding CM2 domain hence the expressed fusion protein contains a single lipoyl domain at the N terminus. Proteins were expressed in *Escherichia coli* (*E. coli*) C41 (DE3) strains in LB broth, and purified using Ni-NTA chromatography. The lipoyl domain was cleaved off the fusion protein with TEV protease, followed by size exclusion chromatography (50 mM Tris-HCl pH7.0, 200 mM NaCl, 5 mM β-mercaptoethanol). The untagged construct contains a GGS motif at the N terminus. Initial protein construct has two lipoyl domains tagged at each terminus, and the final construct, after TEV proteolytic cleavage, contains a GGS motif at the N terminus as well as an EFGENLYFQ motif at the C terminus, which are the remnants of the protease cleavage sites. This protein sample was used to set up the initial crystallization trays, from which the phasing crystals were obtained. In anticipation of the high flexibility of the C-terminal motif, a new construct was designed and cloned with a stop codon included at the 3′ end of the insert (see above). This construct was much more soluble and was used for further crystallization trials, eventually yielding the dataset used for refinement of the CM2 structure.

##### The LZ-CM2 complex

To generate complexes between CM2 and MBP-LZ WT or L535E, the purified CM2 was incubated with excess of either the MBP-LZ WT or L535E in 50 mM Tris pH7.5, 150 mM NaCl, 5 mM β-mercaptoethanol at 4°C for 3 hr. GST-3C protease was then added to remove the His_6_-MBP tag from the resulting complexes (dialyzed into 50 mM Tris-HCl pH7.5, 200 mM NaCl, 5 mM β-mercaptoethanol at 4°C overnight), and was further purified via reverse Ni-NTA chromatography and size exclusion chromatography (50 mM Tris-HCl pH7.5, 150 mM NaCl, 5 mM β-mercaptoethanol).

##### The MBP-PReM protein

The Cnn-PReM (aa403-608) fragment was cloned into a pETM44 (EMBL) vector encoding an N-terminal His_6_-MBP tag. Protein was expressed using *Escherichia coli* (*E. coli*) B834 (DE3) strains in TB broth. Ni-NTA chromatography was carried out to purify the expressed fusion protein, followed by size exclusion chromatography. Purification buffer contains 50 mM Tris-HCl pH7.5, 150 mM NaCl, 5 mM β-mercaptoethanol.

##### The PReM-GFP protein

The Cnn-PReM (aa403-608) fragment was cloned into a custom pWaldo-GFP-A206K vector (point mutation introduced to stop GFP dimerization in solution). This vector encodes a T7 polymerase promoter site and a C-terminal GFP tag followed by 6 Histidine residues. Protein was expressed in *Escherichia coli* (*E. coli*) B834 (DE3) strains in TB broth, and purified using Ni-NTA chromatography followed by size exclusion chromatography (50 mM Tris-HCl pH7.5, 150 mM NaCl, 5 mM β-mercaptoethanol).

#### Crystallization

##### The LZ-CM2 complex

Purified LZ-CM2 protein was dialyzed into 20 mM Tris-HCl pH7.0, 150 mM NaCl, 10 μM ZnCl_2_, 1 mM TCEP at 4°C overnight. For setting up crystallization trials, protein complexes of various lengths were concentrated to 9.18 mg/ml for Cnn_490-544_+CM2, 10.21 mg/ml or 20 mg/ml for Cnn_490-544_ L535E+CM2 and 69.65 mg/ml for Cnn_490-567_+CM2. The commercialized screens from Molecular Dimensions were used for crystallization. The protein concentrations were determined from absorption at 280 nm. Crystals typically grow to their maximal size after 3 days at 21°C in sitting drops, and were fished and flash frozen in liquid nitrogen using ethylene glycol (EG) as a cryo-protectant. LZ-CM2 complexes of various lengths were crystallized in multiple conditions, which are summarized in Table S1. Cnn_490-544_+CM2 and Cnn_490-544_ L535E+CM2 protein complexes crystallized by mixing 300 nL of protein solution with 100 nL of mother liquor; Cnn_490-567_+CM2 crystallized by mixing 150 nL of protein solution with 150 nL of mother liquor.

##### The CM2 apo domain

Purified apo-CM2 protein was dialyzed into 20 mM Tris-HCl pH7.0, 200 mM NaCl, 10 μM ZnCl_2_, 1 mM TCEP at 4°C overnight, and concentrated to 3 mg/ml (construct with C-terminal EFGENLYFQ motif - phasing crystals) or 40 mg/ml (construct lacking above motif – refinement dataset crystals). Crystals of apo-CM2 were grown out either immediately or after overnight at 21°C in sitting drops, and were fished and flash frozen in liquid nitrogen. The crystal, from which the phase information was derived, grew in an optimization screen, using 225 nL protein solution and 75 nL of mother liquor (100 mM HEPES mix (50% pH6.5, 50% pH7.5), 200 mM CaCl_2_, 18% *w/v* PEG6K). 20% *v/v* EG in the mother liquor served as a cryo-protectant. The best diffracting crystal, used for structure refinement, grew in a condition containing 150 nL protein solution and 50 nL of mother liquor (80 mM Sodium cacodylate pH6.5, 160 mM Calcium acetate, 14.4% *w/v* PEG8K/ 20% *v/v* glycerol). The mother liquor itself was used as a cryo-protectant.

#### Data collection and processing

##### The CM2 apo domain

The Cnn-CM2 dataset used for phasing was collected at Diamond beamline I04, at a wavelength of 1.2822 Å. Data were processed using Xia2 pipeline (Winter, 2010) in the 3daii mode (using XDS) (Kabsch, 2010) and AIMLESS (Evans and Murshudov, 2013), and were indexed to space group P6_1_22. A single zinc site was located using Phenix AutoSol (Adams et al., 2010). Phenix Autobuild (Terwilliger et al., 2013) was used for initial modeling, and the structure was refined in Phenix.refine (Adams et al., 2010). Higher resolution data for refinement and rebuilding were later collected at Diamond beamline I04, at 0.9793 Å wavelength in space group P6_1_, with two copies of the initial dimer model placed using Phaser (McCoy et al., 2007). Refinement was then carried out in Phenix.refine (Adams et al., 2010) using TLS refinement, and manual rebuilding was performed in Coot (Emsley and Cowtan, 2004). Metal coordination restraints were generated using ReadySet in Phenix (Adams et al., 2010), to restrain the zinc coordinates. In the final structure, three N-terminal residues (remnants of the protease cleavage site) and eight C-terminal residues (Arg1141-Leu1148) could not be traced due to missing electron density.

##### The LZ-CM2 complex

Initial phasing of the LZ:CM2 was carried out using crystals grown from the LZ_490-552_ protein (CF-1, Table S1). Data were collected at ESRF beamline ID23-1 and processed using the online autoPROC in the spacegroup P2_1_2_1_2_1_ (Vonrhein et al., 2011). Phenix AutoSol initially found seven requested zinc sites (later refined to 2 zincs and 1 iodide). The phases and initial model from this were used to build a more complete model by iteration through Buccaneer (Cowtan, 2006), Refmac (Murshudov et al., 2011) and Coot (Emsley and Cowtan, 2004). The model produced by this process was then used molecular replacement using Phaser (McCoy et al., 2007) or Molrep (Vagin and Teplyakov, 2010) to search the other crystal forms (Table S1, CF-2/3/4). Successful MR solutions were subjected to iterative manual building/refinement using Coot and Phenix.refine. TLS parameters were assessed using the TLSMD server (Painter and Merritt, 2006).

#### SEC-MALS analysis

Samples were dialyzed into 50 mM Tris-HCl pH7.5, 150 mM NaCl, 10 μM ZnCl_2_, 5 mM β-mercaptoethanol. 100 μL of protein sample was injected onto an S75 10/300 column (GE Healthcare). For SEC-MALS analysis of MBP-PReM assemblies (apo or with the addition of CM2 WT or point mutants), 100 μL of protein sample was injected onto a Superose 6 column (GE Healthcare). Light scattering and refractive index were measured using a Dawn Heleos-II light scattering detector and an Optilab-TrEX refractive index monitor. Analysis was carried out using ASTRA 6.1.1.17 software assuming a dn/dc value of 0.186 ml/g.

#### Circular Dichroism

Samples were dialyzed into 10 mM Na_x_H_x_PO_4_ pH 7.5, 0.5 mM TCEP. Buffer subtracted, averaged spectra (4 accumulations) were taken for each sample at 20°C, using a Jasco J-815 instrument. For all the LZ and CM2 constructs, spectra were collected at a protein concentration of 0.2 mg/ml. To further test concentration-dependent folding, additional spectra were collected at 0.6 mg/ml for the LZ WT and point mutant constructs. For the LZ L535E mutant, CD spectra were also collected at 0.8 mg/ml, although the detector was saturated at wavelengths longer than 205 nm and hence the data could not be used for reliable comparison (see Figure S4D).

#### In vitro Cnn network assembly, imaging and analysis

For the experiments in Figure 5D, assembly reactions were set up by mixing purified PReM-GFP and CM2 or its mutant form (I1126E, T1133E and L1137E) at equimolar concentrations (20 μM) in 50 mM Tris-HCl pH7.5, 150 mM NaCl, 5 mM β-mercaptoethanol at room temperature. 0.2 mm red fluorescent polystyrene beads (Invitrogen; pre-blocked with BSA) were added to each reaction at 10,000X dilution to aid in finding the focal plane. 2 μL of each reaction was pipetted, immediately after mixing, onto a non-frosted cover slide, then covered with 18 × 18 mm cover glass (VWR). Networks were visualized using a Zeiss 880 microscope fitted with Airyscan detector using a 63x 1.4NA lens. Images were airyscan-processed in 2D with a strength value of *Auto* (∼6). We used the ‘Analyze Particles’ function of ImageJ to measure the area of Cnn networks, summing the all pixels with intensities above a background threshold. 10 images were taken to generate an average network area for each construct. Fluorescence Recovery After Photobleaching (FRAP) of in vitro Cnn network assemblies was carried out using a Zeiss 880 Airyscan system configured as above. The Cnn network was bleached using 10 iterations of 100% 488 nm laser light on region of interest, and recovery assessed after a 20 min recovery period.

For the experiments in Figure 5G, 10 μM of PReM-GFP pre-incubated with Plk1 kinase (see below) was mixed with 10 μM of CM2 in kinase buffer (50 mM HEPES pH 7.5, 50 mM KCl, 10 mM MgCl_2_, 15 mM EGTA, 20 mM sodium β-glycerophosphate, 0.2 mM ATP, and 1 mM DTT). 0.2 mm red fluorescent polystyrene beads (Invitrogen; pre-blocked with BSA) were added to each reaction at 10,000X dilution to aid in finding the focal plane. 2 μL of total reaction was pipetted onto a non-frosted cover slide, at 3 min time intervals, then covered with 18 × 18 mm cover glass (VWR). Networks were imaged at room temperature using a DeltaVision system (Applied Precision) comprising a wide-field inverted microscope (IX71; Olympus) with 40x/0.95NA Plan Apo objective lens (Olympus) and standard Chroma filter sets. Images were captured using an Evolve EM-CCD camera (Photometrics) and Softworx analysis software (Applied Precision). Images were stitched together and analyzed in ImageJ. Images at the last time point in Plk1 pre-incubated reaction were first thresholded to exclude the background, and this threshold value was then applied to all images at other time points. The same value was used for non-phosphorylated control. We used the ‘Analyze Particles’ function in ImageJ to measure the area of Cnn networks, summing all pixels with intensities above the background threshold. 3 independent experiments were carried out, and the mean value was plotted against time with error bars showing the standard deviation (SD).

#### Antibody production

The anti-pS567-Cnn rabbit antibody was raised against the phospho-peptide: R-R-N-A-M-R-K-A-V-D-R-pS-L-D-L—where pS represents the Ser567 residue that was phosphorylated in the peptide. The Ser567 residue is in the PReM domain and was shown by Mass Spectroscopy to be phosphorylated on Cnn isolated from purified centrosomes but not on Cnn isolated from the cytosol (Conduit et al., 2014a) (unpublished observations). Antibodies were purified against the phospho-peptide and non phospho-specific antibodies were removed by purification against the non-phosphorylated peptide. Peptide production, antibody production and antibody purification was performed by Pocono Rabbit Farm and Laboratory Inc. (USA).

#### In vitro Plk1 kinase assay

20 μM of purified PReM-GFP protein and 0.055 mg/ml Plk1 kinase (ProQinase) was added to kinase buffer in a reaction volume of 20 μl. The reaction was then left at 30°C for 30 min. Plk1 kinase was originally stored in 50 mM HEPES pH 7.5, 100 mM NaCl, 5 mM DTT, 15 mM reduced glutathione, 20% glycerol; therefore, for the non-phosphorylated control equal volume of kinase storage buffer was added to the above reaction instead of Plk1 kinase. The proteins were then analyzed on a dot blot by pipetting 2 μL of 100-fold diluted reaction onto a nitrocellulose membrane (BioRad). The membrane was blocked in PBS + 4% milk + 0.1% Tween20 for 1 hr, and was probed using rabbit anti-Cnn-pS567 antibody (Pocono Rabbit Farm&Laboratory; 1:500 dilution) and anti-rabbit horseradish peroxidase (HRP) (GE Healthcare; 1:3,000 dilution). In order to confirm equal loading of both reactions, the dot blot was also probed using mouse anti-GFP (Roche; 1:500 dilution) and anti-mouse HRP (GE Healthcare; 1:3,000 dilution).

#### 3D-Structured Illumination Microscopy

Embryos were fixed as described (Stevens et al., 2010) and stained using a sheep anti-Cnn antibody at a 1:500 dilution (Cottee et al., 2013) and a rabbit anti-Cnn-pS567 antibody at 1:500 dilution, followed by Alexa 488nm anti-sheep and Alexa 594nm anti-rabbit secondary antibodies at 1:1000 dilution. 3D-SIM microscopy was performed and analyzed as described (Conduit et al., 2014b) on an OMX V3 Blaze microscope (GE Healthcare, UK) with a 60x / 1.42 NA oil UPlanSApo objective (Olympus). The images shown are maximum intensity projections of 17 z-slices. Images from the different color channels were registered with alignment parameters obtained from calibration measurements with 0.2 μm diameter TetraSpeck beads (Life Technologies) using the OMX Editor software.

### Quantification and Statistical Analysis

The statistical details of all experiments are reported in the figure legends and figures, including statistical analysis performed, statistical significance and exact *n* numbers. Statistical significance was assessed using an unpaired t test with Welch’s correction (not assuming equal SDs) in GraphPad Prism (^∗^p < 0.05;^∗∗^p < 0.01;^∗∗∗^p < 0.001;^∗∗∗∗^p < 0.0001). All values are represented as mean ± SD.

### Data and Software Availability

The atomic coordinates of various Cnn LZ-CM2 complexes have been deposited with the accession numbers PDB: 5MVW, 5MW0, 5MW9, 5MWE, and 5I7C.

## Author Contributions

Z.F. led the in vitro biochemical and structural analysis with significant help from S.J., A.F.M.H., M.A.C., and S.M.L. The in vivo analysis was led by A.C. and A.W. with initial help from P.T.C. and J.W.R. All authors contributed to the experimental design and to writing the manuscript.

## Supplemental Information

Table S1. Related to Figures 2, 4, S1, and S3

Table S2. Related to STAR Methods Key Resource Table (Oligonucleotide section)
