# Supplementary material for: Structural Basis for Mitotic Centrosome Assembly in Flies
Source: Cell. 2017 Jun 1;169(6):1078–1089.e13. doi: 10.1016/j.cell.2017.05.030 (PMC5457487; doi:10.1016/j.cell.2017.05.030)
Supplement: Table S1. Related to Figures 2, 4, S1, and S3 [file mmc1.pdf]

Cell, Volume *169*

Supplemental Information

Structural Basis for Mitotic
Centrosome Assembly in Flies

Zhe Feng, Anna Caballe, Alan Wainman, Steven Johnson, Andreas F.M. Haensele, Matthew A. Cottee, Paul T. Conduit, Susan M. Lea, and Jordan W. Raff

Crystal form	CF-1	CF-2	CF-2	CF-3	CF-4	CF-5
**Crystal contents**	**LZ:CM2 WT**	**LZ:CM2 WT**	**LZ:CM2 L535E**	**LZ:CM2 L535E**	**LZ:CM2 WT**	**CM2 WT**
**Construct boundaries**	490-552:1082-1148	490-544:1082-1148	490-544:1082-1148	490-544:1082-1148	490-567:1082-1148	1082-1148
**Crystallization condition**	MD-Morpheus B6 30% EDO-PEG8K, 0.1M Buffer system 2 pH7.5, 0.09M Halogens	20%(w/v) PEG6K, 0.1M Tris pH8.0, 0.2M MgCl2	15%(w/v) PEG4K, 0.2M imidazole malate pH7.0	18%(w/v) PEG2KMME, 0.2M sodium cacodylate pH6.5	20% (w/v) PEG 3350, 0.2M potassium/sodium tartrate	14.4% (w/v) PEG8K, 80mM sodium cacodylate pH6.5, 160mM calcium acetate
**PDB Code**		**PDB: 5MVW**	**PDB: 5MW0**	**PDB: 5MW9**	**PDB: 5MWE**	**PDB: 5I7C**
**Data collection statistics**						
Beamline	ESRF ID23-1	Diamond I04	Petra III P13	Petra III P13	Diamond I03	Diamond I04
Wavelength (Å)	1.282	1.28229	0.97623	0.97623	0.9763	0.9793
Resolution limits (Å)[a]	61.20-2.27 (2.28-2.27)	69.07-1.82 (1.87-1.82)	31.19-2.00 (2.05-2.00)	31.36-2.20 (2.27-2.20)	36.10-2.02 (2.07-2.02)	70.4-2.8 (2.9-2.8)
Space group	$P2_12_12_1$	$P2_1$	$P2_1$	C2	C2	$P6_1$
Unit cell dimensions (Å, °)	33.9,85.6,87.5,90,90, 90	44.9,44.3,72.5,90, 107.8,90	45.8,43.9,68.6,90, 108.3,90	275.8,43.0,48.4,90, 96.6,90	196.0,36.5,43.8,90, 102.8,90	49.2,49.2,211.2,90, 90,120
Unique reflections	12380 (119)	24465 (1806)	17198 (1246)	27935 (2406)	19929 (1450)	7171 (548)
Multiplicity[a]	13.1 (14.1)	16.9 (17.0)	3.0 (3.1)	2.9 (2.9)	3.3 (3.3)	10.4 (10.1)
Completeness (%)[a]	100.0 (100.0)	99.5 (99.8)	97.2 (97.6)	96.4 (96.7)	98.8 (98.7)	99.9 (100.0)
$I/\sigma(I)$[a]	13.1 (2.0)	22.9 (1.1)	6.1 (1.8)	6.7 (1.5)	18.2 (2.1)	19.7 (1.4)
R_{merge} (%)[a,b]	0.13 (1.25)	0.08 (3.05)	0.07 (0.32)	0.07 (0.75)	0.03 (0.62)	0.07 (1.67)
R_{pim} (%)	0.04 (0.34)	0.02 (0.76)	0.05 (0.22)	0.05 (0.53)	0.02 (0.40)	0.02 (0.55)
Processing programs	autoPROC	Xia2	Mosflm/Aimless	Mosflm/Aimless	Xia2	Xia2
**Refinement statistics**						

Resolution limits (Å)		42.74-1.82 (1.89-1.82)	31.19-2.00 (2.13-2.00)	31.35-2.20 (2.29-2.20)	36.10-2.02 (2.13-2.02)	42.6-2.8 (3.2-2.8)
Number of reflections in working set		23200 (2568)	16220 (2656)	26625 (2895)	18985 (2696)	7100 (2235)
Number of reflections in test set		1247 (133)	883 (146)	1240 (167)	941 (132)	369 (137)
R factor of working set[a,c]		0.223 (0.374)	0.237 (0.298)	0.258 (0.374)	0.228 (0.356)	0.243 (0.306)
R_{free}[a,d]		0.246 (0.378)	0.264 (0.336)	0.302 (0.404)	0.269 (0.458)	0.268 (0.392)
Number of atoms (protein/water/other)		1697/208/14	1797/142/1	3651/77/2	1420/26/52	1953/0/2
Residues in Ramachandran favoured region (%)		100	99.1	98.6	98.8	99.6
Ramachandran outliers (%)		0	0.47	0.46	0.59	0
r.m.s.d. bond lengths (Å)		0.005	0.005	0.002	0.005	0.003
r.m.s.d. bond angles (°)		0.559	0.574	0.311	0.568	0.60

[a]Numbers in parentheses refer to the appropriate outer shell.

[b]R_{merge} = 100 x $(\Sigma_{hkl} \Sigma_i |I(hkl;i) - \langle I(hkl) \rangle| / \Sigma_{hkl} \Sigma_i I(hkl;i))$, where $I(hkl;i)$ is the intensity of an individual measurement of a reflection and $\langle I(hkl) \rangle$ is the average intensity of that reflection.

[c]$R_{factor} = (\Sigma_{hkl} ||F_{obs}| - |F_{calc}|| / \Sigma_{hkl} |F_{obs}|)$, where $|F_{obs}|$ and $|F_{calc}|$ are the observed and calculated structure facture amplitudes.

[d]R_{free} equals the *R*-factor of test set (5% of the data removed prior to refinement).

r.m.s.d.: root mean square deviation from ideal geometry.

Table S1.

Related to Figures 2, 4, S1 and S3: Crystallographic data collection and refinement statistics.
