# Supplementary material for: Structural Basis for Mitotic Centrosome Assembly in Flies
Source: Cell. 2017 Jun 1;169(6):1078–1089.e13. doi: 10.1016/j.cell.2017.05.030 (PMC5457487; doi:10.1016/j.cell.2017.05.030)
Supplement: Table S2. Related to STAR Methods Key Resource Table (Oligonucleotide section) [file mmc2.pdf]

Oligonucleotide name	Sequence (5' to 3')
Primers for LZ point mutations	
L528E_F	CATCGATGTCTGCAGTGTGGAGACAAACCGATTGGAAGAGCTG
L528E_R	CAGCTCTTCCAATCGGTTTGTCTCCACACTGCAGACATCGATG
L532E_F	GCAGTGTGCTGACAAACCGAGAGGAAGAGCTGGCTGGTTTCCTC
L532E_R	GAGGAAACCAGCCAGCTCTTCCTCTCGGTTTGTCAGCACACTGC
L535E_F	CTGACAAACCGATTGGAAGAGGAGGCTGGTTTCCTCAACTCTCTG
L535E_R	CAGAGAGTTGAGGAAACCAGCCTCCTCTTCCAATCGGTTTGTCAG
L539E_F	GGAAGAGCTGGCTGGTTTCGAGAACTCTCTGCTGAAGCAC
L539E_R	GTGCTTCAGCAGAGAGTTCTCGAAACCAGCCAGCTCTTCC
L542E_F	CTGGCTGGTTTCCTCAACTCTGAGCTGAAGCACAAAGATGTTCTTG
L542E_R	CAAGAACATCTTTGTGCTTCAGCTCAGAGTTGAGGAAACCAGCCAG
Primers for CM2 point mutations	
H1082C1084EE_F	GCAACTGGTGCAGCCACAGTAGAGGACGAGGCCAAGGTAGATCTTGAAAAC
H1082C1084EE_R	GTTTTCAAGATCTACCTTGGCCTCGTCCTCTACTGTGGCTGCACCAGTTGC
I1126E_F	GCAAAAGCACAAGTTGAGAAAGACGAGAAAAATCAAATACTAAAAACGC
I1126E_R	GCGTTTTTAGTATTTGATTTTTCTCGTCTTTCTCAACTTGTGCTTTTGC
T1133E_F	GACATCAAAAATCAAATACTAAAAGAGCACAATGTGCTGCGAAACGTTCGC
T1133E_R	GCGAACGTTTCGCAGCACATTGTGCTCTTTTAGTATTTGATTTTTGATGTC
L1137E_F	CAAATACTAAAAACGCACAATGTGGAGCGAAACGTTCGCTCAAACATGG
L1137E_R	CCATGTTTGAGCGAACGTTTCGCTCCACATTGTGCGTTTTTAGTATTTG
R1141H_F	CGCACAATGTGCTGCGAAACGTTCATTCAAACATGGAGAATGAGTTATAGG
R1141H_R	CCTATAACTCATTCTCCATGTTTGAATGAACGTTTCGCAGCACATTGTGCG
Primers for LZ fragments	
Cnn490_F	TCCAGGGGCCCATGGATCAACAGAACAGCGCTG
Cnn544_R	CTCGAGTGCGGCCGCTTACTTCAGCAGAGAGTTGAGGAAAC
Cnn552_R	CTCGAGTGCGGCCGCTTACAACACGCCAAGAACATC
Cnn567_R	CTCGAGTGCGGCCGCTTACAAGCTGCGATCCACC
Primers for CM2 fragment	
Cnn1082_F	TACTTCCAGGGTGGATCCCACGACTGTGCCAAGGTAGATC
Cnn1148_R	CAGGTTTTCGCCGAATTCTAACTCATTCTCCATGTTTGAGCG

Table S2.
Related to STAR Methods Key Resource Table (Oligonucleotides section): List of primers used in this paper.
